# Codon Usage Bias Analysis of *Citrus tristeza virus*: Higher Codon Adaptation to *Citrus reticulata* Host

**DOI:** 10.3390/v11040331

**Published:** 2019-04-08

**Authors:** Kajal Kumar Biswas, Supratik Palchoudhury, Prosenjit Chakraborty, Utpal K. Bhattacharyya, Dilip K. Ghosh, Palash Debnath, Chandrika Ramadugu, Manjunath L. Keremane, Ravi K. Khetarpal, Richard F. Lee

**Affiliations:** 1 Advanced Centre for Plant Virology, Division of Plant Pathology, ICAR-Indian Agricultural Research Institute, New Delhi 11012, India; supratik88pc@gmail.com (S.P.); nbu.prosen@yahoo.in (P.C.); ukbhatta2@gmail.com (U.K.B.); 2 ICAR-Central Citrus Research Institute, Nagpur 440033, India; ghoshdk@hotmail.com; 3 Department of Plant Pathology, Assam Agricultural University, Jorhat 785013, India; pnath1@rediffmail.com; 4 Department of Botany and Plant Sciences, University of California, Riverside, CA 92507, USA; chandrika.ramadugu@ucr.edu; 5 National Clonal Germplasm Repository for Citrus & Dates, United States Department of Agriculture-Agricultural Research Service, Riverside, CA 92507, USA; Manjunath.keremane@ars.usda.gov (M.L.K.); rfleevirus@yahoo.com (R.F.L.); 6 Asia-Pacific Association of Agricultural Research Institutions, Bangkok 10100, Thailand; ravi.khetarpal@apaari.org

**Keywords:** *Citrus tristeza virus*, codon usage bias, citrus host, high-frequency codons, natural selection, codon usage adaptation

## Abstract

*Citrus tristeza virus* (CTV), a member of the aphid-transmitted closterovirus group, is the causal agent of the notorious tristeza disease in several citrus species worldwide. The codon usage patterns of viruses reflect the evolutionary changes for optimization of their survival and adaptation in their fitness to the external environment and the hosts. The codon usage adaptation of CTV to specific citrus hosts remains to be studied; thus, its role in CTV evolution is not clearly comprehended. Therefore, to better explain the host–virus interaction and evolutionary history of CTV, the codon usage patterns of the coat protein (*CP*) genes of 122 CTV isolates originating from three economically important citrus hosts (55 isolate from *Citrus sinensis*, 38 from *C. reticulata*, and 29 from *C. aurantifolia*) were studied using several codon usage indices and multivariate statistical methods. The present study shows that CTV displays low codon usage bias (CUB) and higher genomic stability. Neutrality plot and relative synonymous codon usage analyses revealed that the overall influence of natural selection was more profound than that of mutation pressure in shaping the CUB of CTV. The contribution of high-frequency codon analysis and codon adaptation index value show that CTV has host-specific codon usage patterns, resulting in higheradaptability of CTV isolates originating from *C. reticulata* (Cr-CTV), and low adaptability in the isolates originating from *C. aurantifolia* (Ca-CTV) and *C. sinensis* (Cs-CTV). The combination of codon analysis of CTV with citrus genealogy suggests that CTV evolved in *C. reticulata* or other *Citrus* progenitors. The outcome of the study enhances the understanding of the factors involved in viral adaptation, evolution, and fitness toward their hosts. This information will definitely help devise better management strategies of CTV.

## 1. Introduction

*Citrus tristeza virus* (CTV), an aphid-transmitted closterovirus, causes tristeza, a decline in citrus grafted onto CTV-susceptible rootstocks, while some isolates can cause stem pitting on sweet orange and/or grapefruit scions, resulting in reduced fruit production with poor-quality fruit. Tristeza is the most important viral disease of citrus worldwide [1]. Tristeza destroyed over 100 million citrus trees over the last 70 years globally [2]. CTV is a phloem-limited virus having long, flexuous filamentous particles (2000 × 11 nm) consisting of a positive-sense single-stranded RNA (ssRNA) of 19.3kb in length. The genome contains 12 open reading frames (ORFs)—ORF1a and b in the 5′ end half and ORFs 2–11 in 3′ end half—and potentially encodes 19 putative proteins [3]. The ORFs 1a and b encode the replication-related proteins and are translated from the genomic RNA (gRNA), whereas ORFs 2–11 encode proteins p33, p6, p65, p61, p27 (coat protein minor; CPm), p25 (coat protein; CP), p18, p13, p20, and p23, respectively, and are expressed via 3′ co-terminal sub genomic RNAs (sgRNA) [4].

Citrus is cultivated in all geographical zones of India (northeast, northwest, central, and south India), and CTV occurs in nearly all the citrus commercially grown in India [5,6,7]. CTV shows diverse disease syndromes in different citrus species, indicating the occurrence of highly diversified CTV populations in India [7,8]. Extensive genetic diversity in CTV from different citrus-growing regions of India was reported [7,8,9,10,11,12]. At the international level, six distinct CTV genotypes (VT, T36, T30, T3, RB, and T68) and one recombinant entity (HA16-5) were recognized based on phylogenetic analysis of several complete CTV genomes [13,14,15,16].

Several mechanisms are responsible for evolution of CTV; of them, homologous and non-homologous recombination, negative selection, and gene flow are most important [14,15,17]. As the virus is dependent on the host cellular machinery for its translation, the interaction of a virus with a particular host must be studied on the basis of its codon usage pattern. Sixty-one codons encode all 20 amino acids; thus, the degeneracy of genetic code allows more than one codon to encode a single amino acid [18]. Codons encoding the same amino acid are known as synonymous codons. The term codon usage bias (CUB) came in the late 1990s when two to six synonymous codons were found to be responsible for encoding a single amino acid (except methionine and tryptophan) at variable frequencies [19,20] in different organisms. A significant role of CUB in the evolution of viruses was reported [21]. The codon usage pattern of viruses reflects the evolutionary changes that allow the viruses to optimize of their survival and better adapt toward fitness to the external environment and, most importantly, their host [22]. Unfortunately, the studies on CUB and its role in the evolution of plant viruses are limited [23,24,25,26].

Two major models, (i) natural/translational selection and the (ii) mutational/neutral model, explain the codon usage bias [27,28,29]. The natural selection model postulates that there is a co-adaptation of synonymous codon usage and the transfer RNA (tRNA) abundance to optimize translational efficiency, and it is observed in *Saccharomyces cerevisiae* [30] and papillomavirus [31]. The codon choice of some genes would affect the translation of others due to a “shared economy” of the translation apparatus. During the periods of rapid growth, the rate of overall gene expression is limited by the availability of ribosomes. The rate of amino-acid incorporation at more frequent codons occurs at a much higher rate than that of rare codons due to the abundance of the corresponding tRNA species. Previous works suggested that an increase in the translation elongation speed may reduce the number of ribosomes on messenger RNAs (mRNAs) and, therefore, may indirectly increase the rate of initiation of other transcripts due to an increase in the pool of free ribosomes. Thus, the codon usage is adaptive because it enables efficient use of ribosomes and maximizes growth rate of fast growing organisms, such as *Escherichia coli* and *Saccharomyces cerevisiae* [29]. The mutational model postulates that genetic compositional constraints influence the probability of mutational fixation, and this was found in many RNA viruses [23,32,33]. The guanine/cytosine (GC) content is likely to be determined mostly by genome-wide mutation bias rather than by selective forces acting specifically on coding regions. Additional studies demonstrated that codon biases can be statistically predicted in prokaryotes using only intergenic sequences, arguing against the idea of selective forces on coding regions and further supporting the mutational model. However, this model alone cannot fully explain why preferred codons are recognized by more abundant tRNAs [29].

Previously, Cheng et al. [25] carried out comparative analyses of CTV codon usage patterns using the complete genome sequences of 20 CTV isolates obtained from different citrus hosts and studied the codon usage adaptations to *Citrus sinensis* host. They showed that CUB of CTV is low, and it highly resembled the codon usage of *C. sinensis*. However, the codon usage pattern of the CTV isolates with their original citrus hosts was not studied. No reports on codon usage adaptation of CTV to its original citrus hosts are available. The present study reports codon usage adaptations of the coat protein (*CP*) gene of CTV isolates to their respective citrus hosts. Codon usage adaptation varies for different viral genes; the highest degree of codon usage adaptation was observed for those genes expressing at high levels, such as the viral *CP* gene [26,34]. Therefore, in the present study, the *CP* genes of 122 CTV isolates obtained from citrus hosts, *C. aurantifolia* (Mexican lime), *C. reticulata* (mandarin), and *C. sinensis* (sweet orange), were considered for analyzing the synonymous codon usage patterns of CTV. The present study indicates that (i) CTV has overall low CUB, (ii) codon usage adaptations of CTV vary in different citrus hosts with higher adaptation to codon usage pattern of *C. reticulata*, and (iii) codon usage adaptations have a role in the co-evolution of CTV with its host.

## 2. Materials and Methods

### 2.1. Dataset

The complete *CP* gene sequences of 122 CTV isolates worldwide, including 83 Indian isolates originating from three economically important citrus species, *C. aurantifolia*, *C. reticulata*, and *C. sinensis*, obtained from the GenBank database, were used for CUB analysis (Table S1, Supplementary Materials). Of the Indian isolates, the *CP* genes of 79 isolates were reported from the present laboratory (Dr. K. K. Biswas) in the Advanced Center for Plant Virology, ICAR (Indian Agricultural Research Institute), New Delhi [7,9,11,12,15,35]. All 79 isolates were collected from the monoculture practiced citrus (either *C. aurantifolia* or *C. reticulata* or *C. sinensis*) orchard in the particular area. The majorityof citrus orchards surveyed were 30 to 50 years old. Based on source hosts, the CTV isolates were divided into three subgroups: (i) Cs-CTV (originated from *C. sinensis*) of 55 isolates, (ii) Cr-CTV (from *C. reticulata*) of 38 isolates, and (iii) Ca-CTV (from *C. aurantifolia*) of 29 isolates (Table S1, Supplementary Materials). The codon usage data for the three citrus hosts were obtained from the codon usage database (available at https://hive.biochemistry.gwu.edu/review/codon) [36].

### 2.2. Nucleotide Composition Analysis and Effective Number of Codons (ENc)

The overall frequencies of occurrence of nucleotides (A%, U%, C%, and G%), the nucleotide at the third (wobble) position of synonymous codons (A3%, U3%, C3%, and G3%), G+C at the first (GC1), second (GC2), and third (GC3) positions, and G+C at the first and second positions (GC1,2) were calculated for the *CP* gene sequence of each CTV isolate using CodonW version 1.4.2 [37] and a web server http://genomes.urv.es [38]. The ENc values are used to measure the extent of CUB of a gene, and ENc values ranging from 20 to 61 often determine the degree of CUB [39]. The ENc value of a gene at or below 35 indicates strong CUB, whereas the gene having an ENc value of 61 indicates that all synonymous codons are used equally [39].

### 2.3. ENc-GC3 Plot and Neutrality Plot

An ENc-GC3plot was used to investigate the influence of mutation or natural selection on CUB. An ENc-GC3 plot is drawn using the ENc values as the ordinate (*Y*-axis) and the GC3 values as the abscissa (*X*-axis). If mutation is the main force in shaping CUB, the ENc values would lie on or near the standard curve. However, if selection is the main force, the ENc values would lie far lower than the standard curve [39].

A neutrality plot (GC12 vs. GC3) is used to decrypt the mutation and selection factors associated with codon usage. GC12 represents the average of GC1 and GC2; GC3 represents the abundance of G+C at the third codon position. A GC12 vs. GC3 plot is drawn using GC12 as the ordinate (*Y*-axis) and GC3 as the abscissa (*X*-axis). Each dot in the plot represents a *CP* gene of an individual CTV isolate. In neutrality plots, if the correlation between GC12 and GC3 is statistically significant and the slope of the regression line is close to 1(the points positioned on the diagonal line), then mutation pressure is the key factor behind the CUB. Conversely, a lack of correlation between GC12 and GC3 indicates selection against mutation bias [40].

### 2.4. Relative Synonymous Codon Usage (RSCU) and Contribution of High-Frequency Codon (CHFC)

The RSCU value of a codon is the ratio of its observed frequency to its expected frequency given that all codons for a particular amino acid are used equally [27]. RSCU values <1.0, 1.0, and >1.0 represent negative codon usage bias, no bias, and positive bias, respectively. In the present study, a synonymous codon with RSCU values ≥1.05 was referred to as a high-frequency codon. The RSCU values of viruses and hosts were calculated using a previously described method [27] as given in the following equation:

$${\mathrm{RSCU}}_{\mathrm{ij}}=\frac{g_{\mathrm{ij}}}{\sum_{j}^{\mathrm{ni}}g_{\mathrm{ij}}}\times \mathrm{ni}$$

where RSCU_ij_ is the relative synonymous codon usage value of the i-th codon for j-th amino acid, and g_ij_ is the observed number of i-th codon for the j-th amino acid which has an “ni” kind of synonymous codon.

To discriminate the host-preferred high-frequency codon (HFC_H_) from the virus-preferred high-frequency codon (HFC_V_), the RSCU value of genes of CTV was compared with the RSCU value of the potential citrus host. A formula derived from the RSCU equation [27] was used for the quantitative measurement of HFC_H_ and HFC_V_ in the *CP*genes of CTV. In the present study, the equation was termed as “contribution of high-frequency codon” (CHFC), which was calculated using the following formula:

$${\mathrm{RSCU}}_{\mathrm{ij}}=\frac{g_{\mathrm{ij}}}{\sum_{j}^{\mathrm{ni}}g_{\mathrm{ij}}}\times \mathrm{ni};$$

$$\mathrm{or}\;g_{\mathrm{ij}}=\frac{{\mathrm{RSCU}}_{\mathrm{ij}}\times \sum_{j}^{\mathrm{ni}}g_{\mathrm{ij}}}{\mathrm{ni}};$$

$$\mathrm{or}\;{\mathrm{CHFC}}_{j}=\sum_{j}^{{\mathrm{ni}}^{*}}g_{\mathrm{ij}}=\sum_{j}^{{\mathrm{ni}}^{*}}\frac{{\mathrm{RSCU}}_{\mathrm{ij}}\times f_{j}}{\mathrm{ni}}.$$

The contribution of the high-frequency codon for the j-thamino acid (CHFCj) is the summation of the observed number of ni* kinds of synonymous codon for the j-th amino acid; ni* denotes the observed number of high-frequency codons among ni kinds of synonymous codon. The observed frequency of the j-th amino acid (f_j_) is equivalent to the summation of the observed number of ni kind of codon for the j-th amino acid. The frequency of the j-th amino acid (f_j_) was calculated using MEGA 6.0 [41]. 

### 2.5. Codon Adaptation Index (CAI)

Codon adaptation index (CAI) is a quantitative measure that predicts the highest relative adaptation of the viruses to their potential host. CAI is calculated using a web server http://genomes.urv.es/CAIcal/ [38]. CAI values range from 0 to 1. The sequences with higher CAIs are considered to be preferred over those with lower CAIs [38].

### 2.6. Correspondence Analysis (COA)

Correspondence analysis (COA) is a multivariate statistical analysis to establish the relationships between variables and samples. In COA analysis, 59 codons (excluding Met, Trp, and stop codons) represent along 59 orthogonal axes in high-dimension space [42]. RSCU values are plotted in this high-dimension space to study the codon usage patterns. COA analysis was performed using CodonW version 1.4.2 [37].

### 2.7. Statistical Analysis

Correlation analysis among nucleotide composition and the other codon usage indices were performed using SPSS 19.0 (IBM Corp., Armonk, New York, USA). The ENc values of the isolates belonging to different CTV subgroups were analyzed for significant correlation among them using one-way ANOVA in SPSS 19.0.

## 3. Results and Discussion

### 3.1. Preference of G/U-Ended Codon Over A/C-Ended Codon in the AU-Rich CTV CP Gene

To determine the potential influence of compositional constraints on codon usage, the nucleotide compositions of the CTV coding sequences were determined. In the present study, *CP* genes of 122 CTV isolates comprising a total of 81,984 nucleotides were analyzed (Table S2, Supplementary Materials). The mean values of A% (28.73 ± 0.05) and U% (26.99 ± 0.03) were highest, followed by G% (25.90 ± 0.05) and C% (18.38 ± 0.04). The mean values of AU% and GC% were 55.72 ± 0.05 and 44.28 ± 0.05, respectively, whereas the mean values of AU3% and GC3% were 54.75 ± 0.13 and 45.25 ± 0.13, respectively (Table 1). According to the nucleotide occurrence frequencies, CTV *CP* genes are AU-rich. Therefore, A and U seem to be found more commonly than G and C at the wobble position of *CP* gene sequences. However, the nucleotides at wobble positions of synonymous codons (A3, U3, G3, and C3) show that the mean values of U3% (33.98 ± 0.09) and G3% (25.04 ± 0.15) were higher than the mean values of A3% (20.77 ± 0.14) and C3% (20.22 ± 0.09) (Table 1). The uneven usage of A3/U3 and G3/C3 nucleotides in AU-rich *CP* genes in the present study indicates that the compositional patterns of the CTV *CP* genes are more complex than the commonly observed GC- and/or AU-rich compositions of most virus genes. For instance, a GC- or AU-rich genome tends to contain codons preferentially ending with either G/C or A/U. Such trends, when observed, support the influence of mutation pressure. Earlier, Kumar et al. [43] showed the preference of A/U-ended codons over G/C-ended codons in an AU-rich genome and suggested that mutational pressure was the major factor in shaping the codon usage bias of *Equine influenza virus* (EIV). Interestingly, in the CTV *CP* sequence, despite the higher percentage of AU vs. GC, the preferred codons end with U or G, rather than in G/C- or A/U- ended codons. This unequal use of nucleotides suggests the overlapping influences of natural selection and mutational pressure on the codon preferences in the present *CP* gene sequences. Similar trends of unequal use of nucleotides and overlapping influences of natural selection and mutational pressure in the *Zika virus* (ZIKV) genome were shown by Butt et al. [44].

### 3.2. CTVCP Gene Displays Low Codon Usage Bias (CUB) and Higher Genomic Stability

The magnitude of CUB of the *CP* gene of 122 CTV isolates was measured using the effective number of codons (ENc). The ENc values among the present CTV isolates are high and ranged from 48.58 to 59.2 with a mean of 53.88 ± 0.22 (Table 1; Table S2, Supplementary Materials). The higher ENc values in CTV *CP* genes indicate low CUB, resulting in higher genomic stability in CTV. However, the mean ENc values of the three CTV subgroups were calculated as 53.51 for Cr-CTV, 53.74 for Cs-CTV, and 54.63 for Ca-CTV (Table 1). One-way ANOVA analysis showed that there was no significant difference in ENc values among the present CTV subgroups. Previously, analyzing 20 complete CTV genomes, Cheng et al. [25] showed an average ENc value of 53.0 for the CTV genome and suggested no excessive CUB in CTV. Thus, the previous study [25] and this present study indicate that CTV has a lower CUB, resulting in higher genomic stability. The low CUB might be beneficial to CTV on its fitness to the host species with potentially distinct codon preferences. Low CUB was also observed in several RNA viruses, such as *Ebola virus* (Enc: 57.23) [45], *Chikungunya virus* (ENc: 55.56) [22], *Zika virus* (ENc: 53.93) [44], *Hepatitis C virus* (ENc: 52.62) [46], and *Equine influenza virus* (ENc: 52.09) [43]. In an RNA virus population, faster replicators are favored as the virus shares a common resource with the host for their translational machinery [47]. As the RNA-dependent RNA polymerase (RdRP) lacks the 3′–5′ proof-reading activity, a high replication rate sometimes decreases the population fitness by introducing deleterious mutations in the viral genome [47]. A lower replication rate increases the fidelity, which leads to better fitness of the virus population. Thus, a low CUB of RNA viruses has an advantage for efficient replication in the host cells by reducing the competition between the virus and host in using the synthesis machinery [32].

### 3.3. Natural Selection and Mutation Pressure Both Play Roles in Codon Usage Bias of CTV

**Figure 1:**
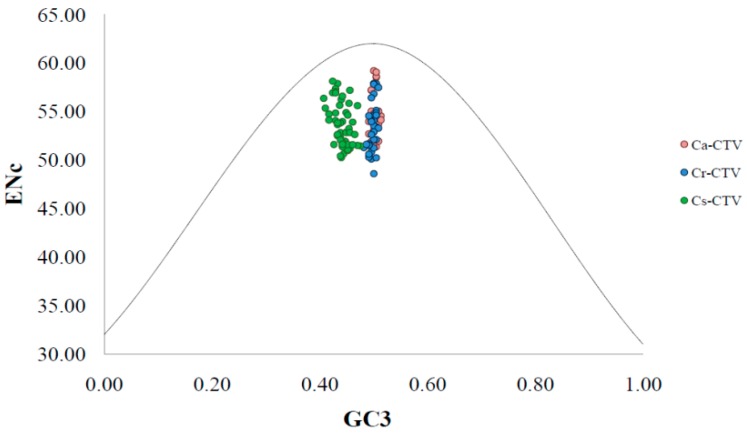
ENc-GC3 plot analysis of the coat protein (*CP*) genes of *Citrus tristeza virus* (CTV) isolates. ENc denotes the effective number of codons, and GC3 denotes the GC content at the third synonymous codon position. The black dotted line represents the expected curve derived from the positions of strains when the codon usage is only determined by the GC3 composition. Different CTV subgroups are indicated as Ca-CTV, Cr-CTV, and Cs-CTV (from *Citrus aurantifolia*, *C. reticulata*, and *C. sinensis*, respectively) with different color markers as shown to the right of the figure.

In the present study, the ENc values of CTV isolates ranged from 48.58 to 59.20 at GC3 values of 0.36–0.41 (Figure 1). All the studied CTV isolates clustered below the standard ENc curve, indicating that CUB of CTV genome is influenced by both the natural selection and the mutational pressure. Earlier, the role of translation/natural and mutational selection on CUB in *Papaya ring spot virus* (PRSV) was reported [26]. It was shown earlier by Adams and Antoniw [23] that mutational pressure has a major role in the CUB of plant viruses. However, the recent report of Chakraborty et al. [26] and the present study show that both the natural selection and mutational pressure have influence on the CUB in plant viruses. On close inspection, in the ENc-GC3 plot, all CTV isolates under Ca-CTV and Cr-CTV subgroups clustered together, whereas the Cs-CTV subgroup clustered separately (Figure 1). This finding indicates differences in the magnitude of natural selection and mutation pressure on CUB among Ca-CTV, Cr-CTV, and Cs-CTV.

### 3.4. Natural Selection Plays Key Role in Shaping the Codon Usage Bias of CTV

**Figure 2:**
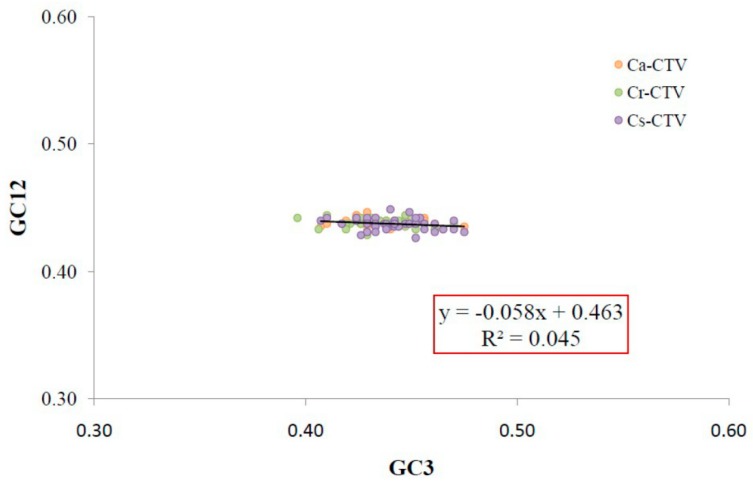
Neutrality plot analysis (GC12 vs. GC3) for the coat protein genes of CTV isolates. GC12 stands for the average value of GC contents at the first and second positions of the codons (GC1 and GC2), while GC3 refers to the GC contents at the thirdposition of the codons. The black line is the linear regression of GC12 against GC3; the regression curve can be described as *y* = −0.058*x* + 0.463, *R*² = 0.045. Different CTV subgroups are indicated with different color markers as shown to the right of the figure.

The magnitude of mutation pressure and natural selection in CUB was investigated by constructing a neutrality plot (GC12 vs. GC3). In the neutrality plot, the slope (−0.058) of the regression line was found to be close to zero (Figure 2), the range of GC3 values were narrow (0.36–0.41), and there was no significant correlation between GC12 and GC3 (Table 2). All the data suggest that natural selection might play a major role in shaping the CUB in the *CP* genes studied. Earlier, the significant role of natural selection in shaping the CUB in *Saccharomyces cerevisiae* [30] and papillomavirus [31] was reported.

### 3.5. Codon Usage Bias Has Significant Correlation with the Nucleotide Compositional Constraint in CTV

**Figure 3:**
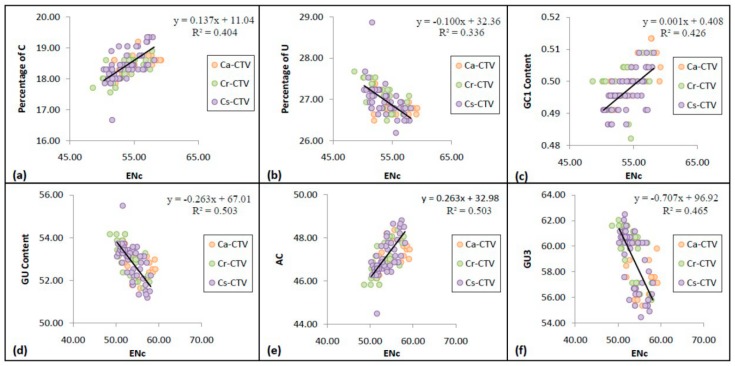
Correlations between effective number of codons (ENc) and the nucleotide composition of CTV coat protein gene. The relationship of ENc with C nucleotide content (**a**), U nucleotide content (**b**), GC1 content (**c**), GU content (**d**), AC content (**e**), and GU3 content (**f**). The regression curves can be described as *y* = 0.137*x* + 11.04, *R*^2^ = 0.404 (**a**); *y* = −0.100*x* + 32.36, *R*^2^ = 0.336 (**b**); *y* = 0.001*x* + 0.408, *R*^2^ = 0.426 (**c**); *y* = −0.236*x* + 67.01, *R*^2^ = 0.503 (**d**); *y* = 0.263*x* + 32.98, *R*^2^ = 0.503 (**e**); *y* = −0.707*x* + 96.92, *R*^2^ = 0.465 (**f**). Different CTV subgroups are indicated with different color markers as shown to the right of the figure.

The relationship between CUB and nucleotide composition was investigated through multivariate correlation analysis (Table 2). In the analysis, ENc showed significant positive correlation with GC1, C, and AC (*r* = 0.610, *r* = 0.614, and *r* = 0.659, respectively, at *p* < 0.01) and significant negative correlation with U, GU, and GU3 (*r* = −0.595, *r* = −0.659, and *r* = −0.646, respectively, at *p* < 0.01) (Figure 3). It suggests that an increasing G/U nucleotide composition may enhance the CUB that is influenced by the nucleotide compositional constraint in CTV. The significant role of nucleotide compositional constraints in shaping the CUB in many RNA and DNA virus genomes was reported [48,49].

### 3.6. Higher Codon Usage Variation in Ca-CTV Subgroup

**Figure 4:**
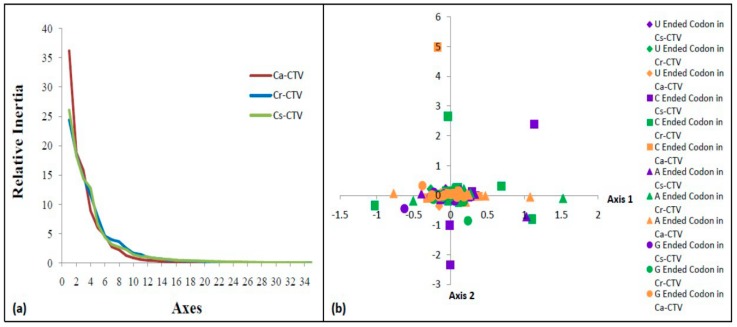
(**a**) Contribution axes based on the correspondence analysis (COA). The relative inertia of 34 axes generated by COA of the relative synonymous codon usage (RSCU) values of CTV subgroup isolates. (**b**) COA analysis of synonymous codon usage patterns: RSCU values of the 59 synonymous codons of three CTV subgroup isolates were plotted in the first two main axes (Axis 1 and Axis 2). Different nucleotide-ended codons of each CTV subgroup isolates are marked in the figure by different colors and shapes.

In correspondence analysis (COA), Axis 1 and Axis 2 were found to be main contributors (two main dimensional coordinates) of codon usage, whereby Axis 1 explained 36.21, 24.40, and 26.08% contribution, and Axis 2 explained 18.79, 18.50, and 18.28% contribution for Ca-CTV, Cr-CTV, and Cs-CTV subgroups, respectively (Figure 4a). Axis 1 explains higher usage of 36.21% for Ca-CTV and lesser usage for both the Cr-CTV (24.4%) and Cs-CTV (26.08%). Therefore, Axis 1 was considered to be the major contributor for codon usage in Ca-CTV, indicating higher codon usage variation in CTV isolates originated from *C. aurantifolia* (Ca-CTV).

### 3.7. G/U-Ended Codons Display Higher Influence on Codon Usage of CTV

In the present COA analysis, the first two axes, Axis 1 and 2, explained half of the total variation, and each subsequent axis explained a declining amount of codon usage variation (Figure 4a). Therefore, the present analysis was restricted to the main axes, Axis 1 and 2. When CTV codons were sorted based on the RSCU values across Axis 1 and 2, the extreme values were occupied by A- and C-ended codons. Also, only the C-ended codons were distributed along Axis 2 (Figure 4b). A separation of codons on the two main axes appears, and it might be largely due to the frequency differences between G/U- and A/C-ended codons. Therefore, the data indicate that G/U-ended codons have higher influence on the codon usage of CTV *CP* genes.

### 3.8. The High-Frequency Codons Are Evolutionarily Conserved in CTV

In the RSCU analysis, 24 high-frequency codons were identified; of them, 13 codons (UUU, CUU, GUU, CCU, ACU, GCU, UAU, UCU, AGU, CGU, UGU, GAU, and GGU) were U-ended, and the remaining codons (UUG, GUG, CCG, AAG, UUA, AUA, AGA, GAA, GUC, CAC, and AAC) wereG/A/C-ended (Table 3). Except for UUU and GUC, all high-frequency codons were found to be conserved in all CTV subgroups (Ca-CTV, Cr-CTV, and Cs-CTV). Interestingly, any bias for the codons encoding glutamine was not detected in any of the CTV subgroups. Thus, the RSCU analysis suggested that most of the high-frequency codons are evolutionarily conserved in the CTV *CP* gene.

### 3.9. The Codon Usage Pattern of Cs-CTV Is Different from That of Ca-and Cr-CTV

More than one high-frequency codon was detected in all three six-fold degenerate amino acids (Leu, Ser, and Arg) in all the CTV *CP* genes studied. Usage of three high-frequency codons (UUA, UUG, and CUU) was observed in leucine, while an equal number of two (UCU and AGU) was observed in serine, with AGA and CGU observed in arginine (Table 3). Among the five four-fold degenerate amino acids (Val, Pro, Thr, Ala, and Gly), only valine and proline showed more than one high-frequency codon: equal numbers of two codons for valine (GUU and GUG) and proline (CCU and CCG). The RSCU analysis showed that all high-frequency codons, except UUU and GUC, were conserved in all CTV subgroups. The UUU codon encoding phenylalanine was found to be conserved in Ca-CTV and Cr-CTV subgroups. Furthermore, valine of all the CTV subgroups had GUU and GUG, but the Cs-CTV subgroup additionally had GUC. These data suggested that the codon usage pattern of CTV isolates originated from *C. sinensis* (Cs-CTV) is different from the codon usage pattern of CTV isolates originated from *C. aurantifolia* (Ca-CTV) and *C. reticulata* (Cr-CTV).

### 3.10. CTV CP Gene Exhibits Higher Codon Usage Bias toward U-Ending Codons

In the present study, nucleotide composition analysis showed that G/U-ending codons are preferred in the CTV *CP* gene. However, RSCU analysis specifically showed that, among the 24 high-frequency codons, 13 codons are U-ended. These data indicate that the CTV *CP* gene exhibits higher CUB toward U-ending codons (Table 3). Previously, Ahmed et al. [50] reported the bias of U/A-ending codons in the genome of the citrus species [50]. Therefore, the preference of U at the wobble position in both the CTV *CP* gene and its host, *Citrus*, indicates a close relationship between virus and host in codon usage pattern.

### 3.11. CTV Is Biased toward Its Host Codon Usage Pattern

As a close relationship in codon usage pattern between CTV with its potential citrus host was found, RSCU analysis was carried out to establish a correlation between the high-frequency codon usage pattern of the CTV *CP* gene and citrus host. In the present study, in RSCU analysis, 15 amino acids showed 18 “host-preferred high-frequency codon” (HFC_H_) usage in all the CTV isolates, and three amino acids (Ile, His, and Gln) showed no HFC_H_ usage (Table 3). In close observation, 15 HFC_H_ codons (UUG, CUU, GUU, GUG, CCU, ACU, GCU, UAU, UCU, AGU, AGA, UGU, GAU, GAA, and GGU) were shown to encode 13 amino acids for Cs-CTV (*C. sinensis*) subgroup, 13 HFC_H_ codons (UUU, UUG, CUU, GUU, GUG, CCU, ACU, GCU, UCU, AGA, AAG, GAU, and GGU) were shown to encode 11 amino acids for Cr-CTV (*C. reticulata*) subgroup, and 12 HFC_H_ codons (UUU, UUG, CUU, GUU, CCU, ACU, GCU, UCU, AGA, AAC, GAU, and GGU) were shown to encode 11 amino acids for Ca-CTV (*C. aurantifolia*) subgroup (Table 3).The present data reveal that all CTV subgroups showed coincident codon usage pattern with their respective citrus hosts, i.e., biased toward the host codon usage pattern, indicating the influence of host translational selection in shaping the codon usage of CTV.

### 3.12. CTV Shows Mixture of Coincident and Antagonistic Codon Usage Patterns to Its Respective Host

In the RSCU analysis, 12 amino acids showed 13 “virus-preferred high-frequency codon” (HFCv) usage in all CTV isolates, and six amino acids (Phe, Thr, Ala, Gln, Asp, and Gly) showed no HFCv usage (Table 3). In close observation, 11 HFC_V_ codons (UUA, AUA, GUG, CCG, UAU, AGU, CGU, UGU, CAC, AAG, and GAA) were shown to encode 11 amino acids for Ca-CTV subgroup, 10 HFC_V_ codons (UUA, AUA, CCG, UAU, AGU, CGU, UGU, CAC, AAC, and GAA) were shown to encode10 amino acids for Cr-CTV subgroup, and eight HFC_V_ codons (UUA, AUA, GUC, CCG, CGU, CAC, AAC, and AAG) were shown to encode eight amino acids for Cs-CTV subgroup (Table 3). These data show an antagonistic codon usage pattern of CTV relative to its host codon usage patterns, indicating that some high-frequency codons of the CTV *CP* gene escape from the host translational selection pressure.

Among the 23 preferred codons including HFC_H_ and HFCv codons identified, the ratio of coincident/antagonist preferred codons was 15/8 between Cs-CTV isolates and *C. sinensis*, 13/10 between Cr-CTV isolates and *C. reticulata*, and 12/11 between Ca-CTV isolates and *C. aurantifolia*. CTV showed no complete coincidence or complete antagonism to any host codon usage pattern. Therefore, CTV evolved as a mixture of coincident and antagonistic codon usage patterns to the respective host, indicating that selection pressure exerted by the host greatly influenced the codon usage pattern of CTV. A similar pattern of mixed codon preferences was also detected in *Chikungunya virus* (CHIKV) and *Zika virus* (ZIKV) with their hosts (*Homo sapiens*) and vectors (*Aedesaegypti* and *Ae. albopictus*) [22,44]. In contrast, viruses like poliovirus and hepatitis A evolved either completely identical or opposite patterns of codon usage to their hosts [51,52]. This is the first study which reports a mixture of coincident and antagonistic codon usage patterns of a plant virus (CTV) to its respective host.

### 3.13. Host-Preferred High-Frequency Codons Exert Greater Effect on the Codon Usage of CTV

**Figure 5:**
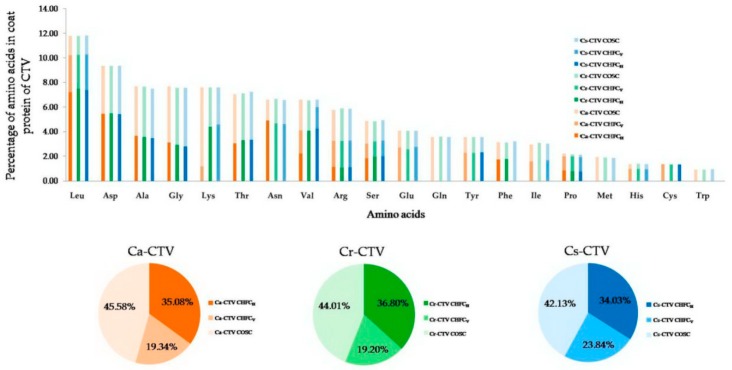
Contribution of high-frequency codons. Bar diagram showing the percentage of 20 amino acids in the coat protein of the three CTV subgroup isolates. Different color stacked bars indicate the percentages of amino acid encoded by the host-preferred high-frequency codon (HFC_H_), virus-preferred high-frequency codon (HFC_V_) and the other synonymous codon (OSC). CHFC_H_, CHFC_V_, and COSC denote contributions of host-preferred high-frequency codon, virus-preferred high-frequency codon, and other synonymous codon. Pie diagram represents the percentages of CHFC_H_, CHFC_V_, and COSC encoded amino acids in coat protein of different CTV subgroups. Different CTV subgroups are indicated with different color markers as shown to the right of the figure.

In the CHFC analysis, four amino acids (Leu, Asp, Asn, and Phe) of Ca-CTV, five amino acids (Leu, Asp, Lys, Val, and Phe) of Cr-CTV, and five amino acids (Leu, Asp, Val, Tyr, and Cys) of Cs-CTV showed usage of HFC_H_ codons (Figure 5 and Table S3, Supplementary Materials). Six amino acids (Pro, Ile, Glu, Tyr, His, and Cys) of Ca-CTV, six amino acids (Asn, Pro, Glu, Tyr, His, and Cys) of Cr-CTV, and six amino acids (Lys, Asn, Pro, Ile, Glu, and His) of Cs-CTV showed usage of HFC_V_ codons (Figure 5 and Table S3, Supplementary Materials). Interestingly, most of the HFC_H_ coded amino acids (Leu, Asp, Lys, and Val) in CTV CP were found to be highly abundant, whereas HFC_V_ coded amino acids (Pro, Glu, Tyr, His, Ile, and Cys) were less abundant (Figure 5). Therefore, HFC_H_ codons exert a greater effect on the CTV codon usage, and this might be beneficial to CTV for fine-tuning the translational efficiency. Earlier, for hepatitis C virus, it was suggested that HFC_H_ (coincident) codon usage between virus and host allowed the corresponding amino acids to be translated efficiently, whereas HFC_v_ (antagonistic) codon usage may allow viral proteins to be folded properly, although the translation efficiency of the corresponding amino acids might be reduced [46].

### 3.14. Varied Degrees of CTV Codon Usage Adaptation to Different Citrus Hosts

In RSCU analysis, a higher number of 15 HFC_H_ codons was found in the Cs-CTV subgroup, followed by 13 in Cr-CTV and 12 in Ca-CTV. However, for HFC_V_ codons, this number was 11 in Ca-CTV, 10 in Cr-CTV, and eight in Cs-CTV. The RSCU analysis suggested that Cs-CTV subgroup has higher codon usage adaptation to its *C. sinensis* host as it has a high number of HFC_H_ codons (15) and a low number of HFC_V_ codons (8). When CHFC analysis was carried out, it was observed that the CHFC_H_ values were 36.80% in Cr-CTV, 35.08% in Ca-CTV, and 34.03% in Cs-CTV subgroups (Figure 5 and Table S3, Supplementary Materials). On the other hand, the CHFC_V_ values were 23.84% in Cs-CTV, 19.34% in Ca-CTV, and 19.20% in Cr-CTV (Figure 5 and Table S3, Supplementary Materials). Thus, CHFC analysis suggests that Cr-CTV subgroup has higher codon usage adaptation to *C. reticulata* host as it has a high CHFC_H_ value (36.80%) and a low CHFC_V_ value (19.20%). The combined RSCU and CHFC analysis showed that Cs-CTV subgroup had a higher number (15) of HFC_H_ codons, but a lower CHFC_H_ value (34.03%) compared to Cr-CTV (36.80%) and Ca-CTV (35.08%). The higher CHFC_H_ value in Cr-CTV subgroup suggests that this subgroup has higher codon usage adaptation to *C. reticulata* host. Thus, RSCU analysis alone could not interpret the results of codon usage adaptations of CTV subgroups to their respective citrus hosts.

### 3.15. Cr-CTV Isolates Display Higher Codon Usage Adaptation to C. reticulata Host

To study the codon usage preferences of CTV subgroups in relation to the codon usage preference of their specific citrus hosts, the codon adaptation index (CAI) was computed. The CAI value of the CTV *CP* gene was 0.816 ± 0.001 for Cr-CTV, 0.778 ± 0.002 for Ca-CTV, and 0.755 ± 0.001 for Cs-CTV subgroups. The higher (0.816 ± 0.001) CAI value in the Cr-CTV subgroup than the Ca- and Cs-CTV subgroups indicated that this subgroup adapted the host codon usage pattern more profoundly than the other subgroups. Wu et al. [53] reported that *C. reticulata* is an evolutionarily primitive species in the genus *Citrus*. Therefore, the primitive association of CTV with *C. reticulata* may have provided a better opportunity to adapt to *C. reticulata* over *C. sinensis* and *C. aurantifolia*. These findings suggest that CTV might have evolved millions of years ago in *C. reticulata* or another *Citrus* progenitor, and later vertically or horizontally transmitted to descended *Citrus* species (*C. sinensis* and *C. aurantifolia*).

### 3.16. The Correlation between Varied Magnitude of High-Frequency Codons and Host–Virus Interactions

CTV is thought to be one of the slowest evolving RNA viruses [54]. This might be due to the occurrence of conserved high-frequency codons in CTV as shown in the present study. This study also found in CHFC analysis that CUB varied among the CTV subgroups, which might be attributed to differential selection of HFC_H_ and HFC_V_. As this study found that Cr-CTV adapted the evolutionary primitive citrus host *C. reticulata* codon usage pattern more profoundly than the other CTV subgroups, RSCU analysis was carried out to identify the fate of HFC_H_ into HFC_V_ in descendant citrus hosts *C. sinensis* and *C. aurantifolia*. In RSCU analysis, four HFC_V_ codons (UAU, AGU, UGU, and GAA) of Cr-CTV were identified to be converted into HFC_H_ codons in Cs-CTV, and one HFC_V_ codon (AAC) was found to be converted into an HFC_H_ codon in Ca-CTV (Table 3). The conversion of HFC_H_ into HFC_V_ was found to be low, because only two HFC_H_ codons (GUG and AAG) of Cr-CTV were found to be converted into HFC_V_ in Ca-CTV and one HFC_H_(AAG) was found to be converted into HFC_V_ in Cs-CTV (Table 3). These data show that the conversion rate of high-frequency codons is high for Cs-CTV and low for Ca-CTV.

The conversion of HFC_H_ into HFC_V_ and viceversa might occur due to the variation of the host tRNA pool. The differential usage of high-frequency codons in CTV subgroups might have an influence on the fitness of the virus population and the host–virus interaction. It is known that *C. sinensis* is more susceptible to CTV than *C. aurantifolia* and *C. reticulata* [7,55], and *C. aurantifolia* is also symptomatic, whereas *C. reticulata* remains symptomless to the virus [55]. The CHFC analysis also showed that adaptability to host codon usage pattern was high in Cr-CTV and low in Ca-CTV and Cs-CTV. Therefore, it is suggested that the variation in host codon usage adaptation in CTV might have a role in the symptom expression and pathogenicity of the virus.

## 4. Conclusions

In summary, our findings revealed that the codon usage bias of CTV *CP* gene is weak, and the influence of natural selection is more profound than that of mutation pressure in shaping codon usage pattern of CTV. The study shows host-specific codon usage pattern and higher codon usage adaptability to the evolutionary primitive citrus, *C. reticulata,* and lower usage in the descendant citrus hosts, *C. sinensis* and *C. aurantifolia*. This finding also suggests that CTV might have co-evolved with *C. reticulata* or another citrus progenitor. The variation in codon usage adaptability of CTV with its citrus host might have a role in host–virus interaction and pathogenicity. The results of this study enhance the understanding of factors involved in viral adaptation, evolution, and fitness toward their hosts. As mixed infections are common in naturally occurring CTV-infected citrus plants, the development of disease-resistant citrus plants is challenging. Hence, better knowledge of the codon usage dynamics of CTV variants in mixed populations will aid in designing synthetic gene constructs or in identifying cross-protecting mild strains to achieve broad-spectrum resistance to CTV in the future.

## Figures and Tables

**Table 1:** Nucleotide (Nt) contents of coat protein (*CP*) genes of *Citrus tristeza virus* (CTV)isolates.

Nt Composition	Ca-CTV	Cr-CTV	Cs-CTV	Overall CTV
**A%**	28.78 (0.10)	28.72 (0.26)	28.71 (0.48)	28.73 (0.05)
**C%**	18.41 (0.36)	18.30 (0.51)	18.42 (0.66)	18.38 (0.04)
**U%**	26.91 (0.09)	27.06 (0.29)	26.98 (0.51)	26.99 (0.03)
**G%**	25.90 (0.15)	25.92 (0.33)	25.88 (0.53)	25.90 (0.05)
**GC** **%**	44.31 (0.53)	44.23 (0.18)	44.30 (0.20)	44.28 (0.05)
**AU** **%**	55.69 (0.91)	55.77 (0.47)	55.70 (0.07)	55.72 (0.05)
**A3%**	21.09 (0.40)	20.64 (0.53)	20.69 (0.65)	20.77 (0.14)
**C3%**	20.26 (0.33)	19.92 (0.49)	20.40 (0.64)	20.22 (0.09)
**U3%**	33.84 (0.24)	34.26 (0.17)	33.86 (0.40)	33.98 (0.09)
**G3%**	24.82 (0.34)	25.18 (0.43)	25.06 (0.59)	25.04 (0.15)
**GC3%**	45.07 (0.61)	45.10 (0.32)	45.45 (0.25)	45.25 (0.13)
**GA3%**	45.91 (0.58)	45.82 (0.23)	45.75 (0.19)	45.81 (0.06)
**GU3%**	58.65 (1.08)	59.43 (0.65)	58.91(0.32)	59.01 (0.20)
**AU3%**	54.93 (0.92)	54.90 (0.51)	54.55(0.19)	54.75 (0.13)
**CU3%**	54.09 (0.86)	54.18 (0.44)	54.25 (0.08)	54.19 (0.06)
**GC1**	0.50 (0.00)	0.50 (0.00)	0.50 (0.00)	0.50 (0.00)
**GC2**	0.38 (0.00)	0.38 (0.00)	0.38 (0.00)	0.38 (0.00)
**GC3**	0.44 (0.00)	0.44 (0.00)	0.44 (0.00)	0.44 (0.00)
**GC12**	0.44 (0.00)	0.44 (0.01)	0.44 (0.01)	0.44(0.01)
**CBI**	0.05 (0.01)	0.04 (0.00)	0.04 (0.00)	0.05 (0.00)
**Fop**	0.45 (0.00)	0.45 (0.00)	0.45 (0.00)	0.45 (0.00)
**ENc**	54.63 (0.46)	53.51 (0.39)	53.74 (0.30)	53.88 (0.22)
**L_sym**	216.69 (0.08)	216.76 (0.08)	216.78 (0.07)	216.75 (0.04)
**L_aa**	223.00 (0.00)	223.00 (0.00)	223.00 (0.00)	223.00 (0.00)
**Gravy**	−0.48 (0.00)	−0.49 (0.00)	−0.48 (0.00)	−0.48 (0.00)
**Aromo**	0.08 (0.00)	0.08 (0.00)	0.08 (0.00)	0.08 (0.00)

**Note:** Ca-CTV, Cr-CTV, and Cs-CTV denote the CTV isolates originated from *Citrusaurantifolia*, *C. reticulata*, and *C. sinensis*, respectively. Values within parentheses indicate ± standard errors. A%, U%, C%, and G% represent the overall frequencies of adenine (A), cytosine (C), uracil (U), and guanine (G) nucleotides of the CTV *CP* gene; A3%,U3%, C3%, and G3% represent the nucleotide frequency at the third codon position; GC and AU denote the percentage of G+C and A+U; GC1, GC2, and GC3 denote the G+C at the first, second, and third synonymous codon positions; GA3, GU3, AU3, and CU3 represent the G+A, G+U, A+U, and C+U percentages at the third codon position; CBI, codon bias index, measures the extent to which a gene uses a subset of optimal codons; Fop, frequency of optimum codons, is the ratio of optimal codons to synonymous codons; ENc, effective number of codons; L_sym, number of synonymous codons; L_aa, number of translatable codons; Gravy, general average hydropathicity; Aromo, frequency of aromatic amino acids in hypothetically translated gene product.

**Table 2:** Correlation analysis among different nucleotides compositions of CTV.

	CAI	CBI	Fop	ENc	GC1	GC2	GC12	GC3	A	C	U	G	GU	AC	GU3	AU3
CAI																
CBI	0.820 **															
Fop	0.826 **	0.998 **														
ENc	0.487 **	0.454 **	0.435 **													
GC1	0.387 **	0.247 **	0.233 **	0.610 **												
GC2	−0.278 **	−0.178 *	−0.172	0.047	0.056											
GC12	0.151	0.096	0.088	0.512 **	0.827 **	0.608 **										
GC3	−0.563 **	−0.412 **	−0.402 **	−0.373 **	−0.329 **	0.093	−0.210 *									
A	0.733 **	0.704 **	0.696 **	0.500 **	0.191 *	−0.268 **	0.001	−0.795 **								
C	0.425 **	0.665 **	0.653 **	0.614 **	0.382 **	0.090	0.355 **	0.005	0.388 **	−0.845 **						
U	−0.393 **	−0.506 **	−0.504 **	−0.595 **	−0.426 **	−0.175	−0.438 **	−0.087	−0.358 **	−0.537 **	0.313 **					
G	−0.704 **	−0.778 **	−0.764 **	−0.514 **	−0.182 *	0.273 **	0.009	0.741 **	−0.931 **	−0.788 **	0.686 **	0.906 **				
GU	−0.715 **	−0.822 **	−0.810 **	−0.659 **	−0.329 **	0.131	−0.188 *	0.529 **	−0.874 **	0.788 **	−0.686 **	−0.906 **	−0.471 **			
AC	0.715 **	0.822 **	0.810 **	0.659 **	0.329 **	−0.131	0.188 *	−0.529 **	0.874 **	−0.725 **	0.605 **	0.873 **	0.938 **	−0.938 **		
GU3	−0.749 **	−0.836 **	−0.821 **	−0.646 **	−0.282 **	0.118	−0.158	0.521 **	−0.831 **	0.000	0.097	−0.748 **	−0.530 **	0.530 **	−1.000 **	
AU3	0.568 **	0.431 **	0.420 **	0.370 **	0.325 **	−0.090	0.208 *	−0.996 **	0.793 **	0.000	−0.097	0.748 **	0.530 **	−0.530 **	0.526 **	−1.000 **

** Correlation is significant at the 0.01 level (two-tailed), highlighted with orange color; * correlation is significant at the 0.05 level (two-tailed).

**Table 3:** The relative synonymous codon usage (RSCU) patterns of CTV isolates and their respective citrus hosts.

AA	Codon	Ca-CTV	Cr-CTV	Cs-CTV	*C. aurantifolia (Ca)*	*C. reticulata (Cr)*	*C. sinensis (Cs)*
Phe	UUU	**1.11**	**1.14**	1.01	**1.20**	**1.08**	**1.24**
	UUC	0.89	0.86	0.99	0.80	0.92	0.76
Leu	UUA	**1.54**	**1.40**	**1.47**	0.95	0.88	0.87
	UUG	**2.54**	**2.70**	**2.62**	**1.49**	**1.52**	**1.56**
	CUU	**1.13**	**1.12**	**1.13**	**1.40**	**1.28**	**1.48**
	CUC	0.01	0.01	0.01	**1.16**	0.91	0.71
	CUA	0.24	0.26	0.31	0.48	0.43	0.57
	CUG	0.54	0.50	0.46	0.52	0.98	0.82
Ile	AUU	0.91	0.81	0.80	**1.39**	**1.48**	**1.51**
	AUC	0.50	0.59	0.55	0.93	0.90	0.71
	AUA	**1.59**	**1.6**	**1.65**	0.68	0.63	0.77
Val	GUU	**1.34**	**1.37**	**1.35**	**1.57**	**1.34**	**1.65**
	GUC	1.00	1.01	**1.07**	0.90	0.84	0.63
	GUA	0.51	0.48	0.37	0.55	0.54	0.66
	GUG	**1.15**	**1.13**	**1.21**	0.98	**1.29**	**1.06**
Pro	CCU	**1.49**	**1.44**	**1.38**	**1.84**	**1.54**	**1.50**
	CCC	0.03	0.04	0.06	0.66	0.69	0.58
	CCA	0.37	0.22	0.28	**1.18**	**1.29**	**1.46**
	CCG	**2.11**	**2.30**	**2.28**	0.32	0.49	0.45
Thr	ACU	**1.72**	**1.86**	**1.84**	**1.20**	**1.49**	**1.48**
	ACC	0.89	0.78	0.77	0.99	0.84	0.75
	ACA	0.66	0.64	0.65	**1.34**	**1.21**	**1.36**
	ACG	0.73	0.72	0.73	0.47	0.45	0.42
Ala	GCU	**1.90**	**1.86**	**1.85**	**1.88**	**1.41**	**1.65**
	GCC	0.76	0.82	0.87	**1.08**	1.01	0.69
	GCA	0.88	0.84	0.83	0.78	**1.13**	**1.34**
	GCG	0.47	0.48	0.44	0.25	0.46	0.32
Tyr	UAU	**1.29**	**1.28**	**1.29**	0.94	1.03	**1.23**
	UAC	0.71	0.72	0.71	**1.06**	0.97	0.77
Ser	UCU	**2.27**	**2.42**	**2.42**	**1.68**	**1.41**	**1.56**
	UCC	0.71	0.56	0.53	0.96	0.84	0.72
	UCA	0.75	0.84	0.81	**1.20**	**1.33**	**1.45**
	UCG	0.38	0.32	0.30	0.48	0.69	0.42
	AGU	**1.45**	**1.57**	**1.53**	0.80	0.87	**1.07**
	AGC	0.44	0.29	0.41	0.89	0.85	0.77
Arg	AGA	**1.18**	**1.09**	**1.12**	**2.38**	**1.84**	**1.98**
	AGG	0.69	0.75	0.76	0.83	**1.29**	**1.49**
	CGU	**2.18**	**2.21**	**2.21**	0.89	0.91	0.79
	CGC	0.55	0.49	0.53	0.49	0.70	0.48
	CGA	0.95	1.01	0.92	0.89	0.69	0.70
	CGG	0.45	0.44	0.47	0.52	0.56	0.55
Cys	UGU	**2.00**	**1.95**	**1.95**	0.97	0.76	**1.13**
	UGC	0.00	0.05	0.05	1.03	**1.24**	0.87
His	CAU	0.59	0.62	0.61	**1.15**	**1.05**	**1.32**
	CAC	**1.41**	**1.38**	**1.39**	0.85	0.95	0.68
Gln	CAA	1.01	1.04	1.02	1.02	1.00	1.10
	CAG	0.99	0.96	0.98	0.98	1.00	0.90
Asn	AAU	0.52	0.59	0.59	0.83	**1.11**	**1.30**
	AAC	**1.48**	**1.41**	**1.41**	**1.17**	0.89	0.70
Lys	AAA	0.84	0.84	0.79	**1.05**	0.93	0.99
	AAG	**1.16**	**1.16**	**1.21**	0.95	**1.07**	1.01
Asp	GAU	**1.16**	**1.17**	**1.16**	**1.29**	**1.33**	**1.42**
	GAC	0.84	0.83	0.84	0.71	0.67	0.58
Glu	GAA	**1.32**	**1.26**	**1.35**	1.04	0.99	**1.10**
	GAG	0.68	0.74	0.65	0.96	1.01	0.90
Gly	GGU	**1.62**	**1.54**	**1.48**	**1.26**	**1.09**	**1.20**
	GGC	0.65	0.73	0.75	**1.11**	0.98	0.73
	GGA	0.76	0.74	0.77	**1.13**	**1.21**	**1.29**
	GGG	0.97	1.00	0.99	0.50	0.71	0.77
Trp	UGG	1.00	1.00	1.00	1.00	1.00	1.00
Met	AUG	1.00	1.00	1.00	1.00	1.00	1.00

AA represents the three-letter abbreviation code of 20amino acids. Ca-CTV, Cr-CTV, and Cs-CTV denote the CTV isolates originated from *C. aurantifolia*, *C. reticulata*, and *C. sinensis*, respectively. RSCU values of high-frequency codons/abundantly used codons of the virus and its host are marked in bold. Host-preferred high-frequency codons (HFC_H_) of CTV showing a coincident relationship with host codons are highlighted with gray color, and the virus-preferred high-frequency codons (HFC_V_) of CTV showing an antagonist relationship with host codons are highlighted with green color.

## Supplementary Materials

The following are available online at https://www.mdpi.com/1999-4915/11/4/331/s1: Table S1: Source of CTV isolates originated in different citrus species used in the present study; Table S2: Nucleotide contents of CP genes of 122 CTV isolates; Table S3: Contribution of host-preferred high-frequency codons, virus-preferred high-frequency codons, and other synonymous codons in coat protein of CTV isolates.
