# Supplementary material for: Codon Usage Bias Analysis of Citrus tristeza virus: Higher Codon Adaptation to Citrus reticulata Host
Source: Viruses. 2019 Apr 8;11(4):331. doi: 10.3390/v11040331 (PMC6521185; doi:10.3390/v11040331)
Supplement: Supplementary file 1 [file viruses-11-00331-s001.pdf]

Table S1. Source of CTV isolates originated in different citrus species used in the present study.

SN	Country of origin	Source host	CTV Subgroup	CTV Isolates	Accession No.
1	India	*Citrus aurantifolia*	Ca-CTV	D3	FJ001821
2	India	*Citrus aurantifolia*	Ca-CTV	D4	FJ001822
3	India	*Citrus aurantifolia*	Ca-CTV	CTV-D	AF501867
4	India	*Citrus aurantifolia*	Ca-CTV	Kpg1	GQ392062
5	India	*Citrus aurantifolia*	Ca-CTV	Kpg2	GQ228821
6	India	*Citrus aurantifolia*	Ca-CTV	K4	GQ475541
7	India	*Citrus aurantifolia*	Ca-CTV	K5	KC694140
8	India	*Citrus aurantifolia*	Ca-CTV	K6	GQ475542
9	India	*Citrus aurantifolia*	Ca-CTV	K7	GQ475543
10	India	*Citrus aurantifolia*	Ca-CTV	K9	KC694141
11	India	*Citrus aurantifolia*	Ca-CTV	K10	KC694145
12	India	*Citrus aurantifolia*	Ca-CTV	K18	GQ475549
13	India	*Citrus aurantifolia*	Ca-CTV	K20	GQ475550
14	India	*Citrus aurantifolia*	Ca-CTV	K22	GQ475551
15	India	*Citrus aurantifolia*	Ca-CTV	K23	GQ475552
16	India	*Citrus aurantifolia*	Ca-CTV	AR2	KC590489
17	India	*Citrus aurantifolia*	Ca-CTV	N1	KC986384
18	India	*Citrus aurantifolia*	Ca-CTV	P2	GQ475564
19	India	*Citrus aurantifolia*	Ca-CTV	P3	GQ475565
20	India	*Citrus aurantifolia*	Ca-CTV	P4	GQ475566
21	India	*Citrus aurantifolia*	Ca-CTV	P13	GQ475569
22	India	*Citrus aurantifolia*	Ca-CTV	TP4	KC590502
23	India	*Citrus aurantifolia*	Ca-CTV	TP5	KC590503
24	India	*Citrus aurantifolia*	Ca-CTV	TP9	KC590506
25	India	*Citrus aurantifolia*	Ca-CTV	CTV-B	AF501867
26	China	*Citrus aurantifolia*	Ca-CTV	ML12	EF028325
27	China	*Citrus aurantifolia*	Ca-CTV	ML-13	EF028326
28	China	*Citrus aurantifolia*	Ca-CTV	HB1	EF028324
29	New Zealand	*Citrus aurantifolia*	Ca-CTV	NZRB-M17	FJ525435
30	India	*Citrus reticulata*	Cr-CTV	Kpg3	HM573451
31	India	*Citrus reticulata*	Cr-CTV	K2	GQ475540
32	India	*Citrus reticulata*	Cr-CTV	K8	GQ475544
33	India	*Citrus reticulata*	Cr-CTV	K11	GQ475545
34	India	*Citrus reticulata*	Cr-CTV	K13	KC694142
35	India	*Citrus reticulata*	Cr-CTV	K16	GQ475547
36	India	*Citrus reticulata*	Cr-CTV	K17	GQ475548
37	India	*Citrus reticulata*	Cr-CTV	K24	GQ475553
38	India	*Citrus reticulata*	Cr-CTV	K27	GQ475554
39	India	*Citrus reticulata*	Cr-CTV	K30	GU350407
40	India	*Citrus reticulata*	Cr-CTV	K31	GQ475555
41	India	*Citrus reticulata*	Cr-CTV	K33	GQ475556
42	India	*Citrus reticulata*	Cr-CTV	K37	KY567992
43	India	*Citrus reticulata*	Cr-CTV	K38	KY567993
44	India	*Citrus reticulata*	Cr-CTV	K39	KY567994
45	India	*Citrus reticulata*	Cr-CTV	K40	KY567995
46	India	*Citrus reticulata*	Cr-CTV	K41	KY567996
47	India	*Citrus reticulata*	Cr-CTV	K42	KY567997
48	India	*Citrus reticulata*	Cr-CTV	ARP1	LN997804
49	India	*Citrus reticulata*	Cr-CTV	AR13	KC590490
50	India	*Citrus reticulata*	Cr-CTV	AG26	KC590497
51	India	*Citrus reticulata*	Cr-CTV	AG28	KC590498
52	India	*Citrus reticulata*	Cr-CTV	MB7	KC590496
53	India	*Citrus reticulata*	Cr-CTV	Mnp1	KU530134
54	India	*Citrus reticulata*	Cr-CTV	Mnp2	KU530135
55	India	*Citrus reticulata*	Cr-CTV	Mnp3	KU530136
56	India	*Citrus reticulata*	Cr-CTV	P10	GQ475568
57	India	*Citrus reticulata*	Cr-CTV	KAT5	GQ47561
58	India	*Citrus reticulata*	Cr-CTV	KAT6	GQ47562
59	India	*Citrus reticulata*	Cr-CTV	B165	EU076703
60	Pakistan	*Citrus reticulata*	Cr-CTV	179	HQ329197
61	Thailand	*Citrus reticulata*	Cr-CTV	A18	JQ798289
62	Florida	*Citrus reticulata*	Cr-CTV	FS701-VT	KC517494
63	Florida	*Citrus reticulata*	Cr-CTV	FS701-T30	KC517489
64	Florida	*Citrus reticulata*	Cr-CTV	FS701-T36	KC517486
65	Florida	*Citrus reticulata*	Cr-CTV	FS703-VT	KC517492
66	Florida	*Citrus reticulata*	Cr-CTV	FS703-T30	KC517491
67	Florida	*Citrus reticulata*	Cr-CTV	FS703-T36	KC517487
68	India	*Citrus sinensis*	Cs-CTV	D5	GQ475538
69	India	*Citrus sinensis*	Cs-CTV	D6	FJ001823
70	India	*Citrus sinensis*	Cs-CTV	D7	FJ001824
71	India	*Citrus sinensis*	Cs-CTV	D8	GQ475539
72	India	*Citrus sinensis*	Cs-CTV	D10	FJ001826
73	India	*Citrus sinensis*	Cs-CTV	D11	FJ001827
74	India	*Citrus sinensis*	Cs-CTV	D12	FJ001828
75	India	*Citrus sinensis*	Cs-CTV	D13	FJ001829
76	India	*Citrus sinensis*	Cs-CTV	D14	FJ001830

77	India	*Citrus sinensis*	Cs-CTV	D15	FJ001831
78	India	*Citrus sinensis*	Cs-CTV	AR16	KC590491
79	India	*Citrus sinensis*	Cs-CTV	MB3	KC590492
80	India	*Citrus sinensis*	Cs-CTV	P7	GQ475567
81	India	*Citrus sinensis*	Cs-CTV	P14	GQ47570
82	India	*Citrus sinensis*	Cs-CTV	KAT1	GQ47557
83	India	*Citrus sinensis*	Cs-CTV	KAT2	GQ47558
84	India	*Citrus sinensis*	Cs-CTV	KAT3	GQ47559
85	India	*Citrus sinensis*	Cs-CTV	KAT4	GQ47560
86	India	*Citrus sinensis*	Cs-CTV	KAT7	KC694143
87	India	*Citrus sinensis*	Cs-CTV	CTV-N	AF501870
88	India	*Citrus sinensis*	Cs-CTV	DKG1	AF501869
89	India	*Citrus sinensis*	Cs-CTV	TP1	EF460127
90	India	*Citrus sinensis*	Cs-CTV	TP3	KC590501
91	India	*Citrus sinensis*	Cs-CTV	TP6	KC590504
92	India	*Citrus sinensis*	Cs-CTV	TP7	KC590505
93	India	*Citrus sinensis*	Cs-CTV	TP10	KC590507
94	India	*Citrus sinensis*	Cs-CTV	TP11	KC590508
95	India	*Citrus sinensis*	Cs-CTV	TP12	KC590509
96	Angola	*Citrus sinensis*	Cs-CTV	F5	DQ660346
97	Angola	*Citrus sinensis*	Cs-CTV	O2	DQ660348
98	Angola	*Citrus sinensis*	Cs-CTV	O6	DQ660349
99	Angola	*Citrus sinensis*	Cs-CTV	O7	DQ660350
100	Angola	*Citrus sinensis*	Cs-CTV	P2	DQ660351
101	Angola	*Citrus sinensis*	Cs-CTV	P9	DQ660352
102	Angola	*Citrus sinensis*	Cs-CTV	Q4	DQ660353
103	Angola	*Citrus sinensis*	Cs-CTV	Q8	DQ660354
104	Angola	*Citrus sinensis*	Cs-CTV	Q14	DQ660355
105	China	*Citrus sinensis*	Cs-CTV	S4	EF063109
106	China	*Citrus sinensis*	Cs-CTV	CT14A	JQ911663
107	China	*Citrus sinensis*	Cs-CTV	CT11A	JQ061137
108	China	*Citrus sinensis*	Cs-CTV	AT-1	JQ911664
109	Israel	*Citrus sinensis*	Cs-CTV	VT	U56902
110	New Zealand	*Citrus sinensis*	Cs-CTV	NZ-B18	FJ525436
111	Pakistan	*Citrus sinensis*	Cs-CTV	108	HQ329188
112	Pakistan	*Citrus sinensis*	Cs-CTV	109	HQ329189
113	Pakistan	*Citrus sinensis*	Cs-CTV	135	HQ329191
114	Pakistan	*Citrus sinensis*	Cs-CTV	142	HQ329192
115	Pakistan	*Citrus sinensis*	Cs-CTV	143	HQ329193
116	Pakistan	*Citrus sinensis*	Cs-CTV	146	HQ329194
117	Pakistan	*Citrus sinensis*	Cs-CTV	159	HQ329195
118	Pakistan	*Citrus sinensis*	Cs-CTV	160	HQ329196
119	Spain	*Citrus sinensis*	Cs-CTV	SYR-A2	FN667582
120	Florida	*Citrus sinensis*	Cs-CTV	T3	KC525952
121	Florida	*Citrus sinensis*	Cs-CTV	FL202-VT	KC517493
122	Florida	*Citrus sinensis*	Cs-CTV	FS674-T36	KC517485

Table S2. Nucleotide contents of CP genes of 122 CTV isolates.

CTV Subgroup	Isolates	CAI	CBI	Fop	Nc	GC1	GC2	GC12	GC3	L_sym	L_aa	Gravy	Aromo	length	%A	%C	%U	%G	%GC	%GA	%GU	%AU	%AC	%CU	%A1	%C1	%U1	%G1	%GA1	%GU1	%AU1	%CU1	%A2	%C2	%U2	%G2	%GA2	%GU2	%AU2	%CU2	%A3	%C3	%U3	%G3	%GA3	%GU3	%AU3	%CU3
Ca-CTV	D3	0.23	0.02	0.43	51.38	0.50	0.38	0.44	0.45	217.00	223.00	−0.48	0.08	672.00	28.27	18.01	27.23	26.49	44.49	54.76	53.72	55.51	46.28	45.24	29.91	14.73	20.54	34.82	64.73	55.36	50.45	35.27	35.71	20.09	26.34	17.86	53.57	44.20	62.05	46.43	19.20	19.20	34.82	26.79	45.98	61.61	54.02	54.02
Ca-CTV	D4	0.23	0.02	0.43	51.37	0.50	0.38	0.44	0.45	217.00	223.00	−0.47	0.08	672.00	28.13	18.01	27.23	26.64	44.64	54.76	53.87	55.36	46.13	45.24	29.46	14.73	20.54	35.27	64.73	55.80	50.00	35.27	35.71	20.09	26.34	17.86	53.57	44.20	62.05	46.43	19.20	19.20	34.82	26.79	45.98	61.61	54.02	54.02
Ca-CTV	CTV-D	0.23	0.03	0.44	53.98	0.50	0.38	0.44	0.45	216.00	223.00	−0.49	0.08	672.00	28.42	18.45	26.79	26.34	44.79	54.76	53.13	55.21	46.88	45.24	29.46	15.18	20.09	35.27	64.73	55.36	49.55	35.27	36.16	20.09	26.34	17.41	53.57	43.75	62.50	46.43	19.64	20.09	33.93	26.34	45.98	60.27	53.57	54.02
Ca-CTV	Kpg1	0.22	0.02	0.44	54.55	0.50	0.38	0.44	0.45	216.00	223.00	−0.48	0.08	672.00	28.42	18.45	26.79	26.34	44.79	54.76	53.13	55.21	46.88	45.24	29.91	15.18	20.09	34.82	64.73	54.91	50.00	35.27	35.71	20.09	26.34	17.86	53.57	44.20	62.05	46.43	19.64	20.09	33.93	26.34	45.98	60.27	53.57	54.02
Ca-CTV	Kpg2	0.24	0.08	0.47	53.76	0.50	0.38	0.44	0.41	217.00	223.00	−0.46	0.07	672.00	29.61	18.60	27.08	24.70	43.30	54.32	51.79	56.70	48.21	45.68	29.91	14.73	20.54	34.82	64.73	55.36	50.45	35.27	35.71	20.09	26.34	17.86	53.57	44.20	62.05	46.43	23.21	20.98	34.38	21.43	44.64	55.80	57.59	55.36
Ca-CTV	K4	0.25	0.07	0.47	58.53	0.51	0.38	0.44	0.43	217.00	223.00	−0.48	0.08	672.00	28.87	18.60	26.79	25.74	44.35	54.61	52.53	55.65	47.47	45.39	28.57	14.73	20.54	36.16	64.73	56.70	49.11	35.27	36.16	20.54	25.89	17.41	53.57	43.30	62.05	46.43	21.88	20.54	33.93	23.66	45.54	57.59	55.80	54.46
Ca-CTV	K5	0.25	0.08	0.47	57.18	0.50	0.38	0.44	0.42	217.00	223.00	−0.48	0.08	672.00	29.46	18.45	26.79	25.30	43.75	54.76	52.08	56.25	47.92	45.24	29.02	14.73	20.54	35.71	64.73	56.25	49.55	35.27	36.16	20.09	26.34	17.41	53.57	43.75	62.50	46.43	23.21	20.54	33.48	22.77	45.98	56.25	56.70	54.02
Ca-CTV	K6	0.25	0.06	0.46	54.56	0.50	0.38	0.44	0.41	216.00	223.00	−0.50	0.08	672.00	29.91	18.15	26.93	25.00	43.15	54.91	51.93	56.85	48.07	45.09	29.46	14.29	20.98	35.27	64.73	56.25	50.45	35.27	36.16	20.09	26.34	17.41	53.57	43.75	62.50	46.43	24.11	20.09	33.48	22.32	46.43	55.80	57.59	53.57
Ca-CTV	K7	0.25	0.08	0.47	57.80	0.51	0.38	0.44	0.43	217.00	223.00	−0.46	0.08	672.00	28.72	18.45	26.93	25.89	44.35	54.61	52.83	55.65	47.17	45.39	28.13	14.73	20.54	36.61	64.73	57.14	48.66	35.27	36.16	20.09	26.34	17.41	53.57	43.75	62.50	46.43	21.88	20.54	33.93	23.66	45.54	57.59	55.80	54.46
Ca-CTV	K9	0.25	0.07	0.46	55.03	0.50	0.38	0.44	0.43	216.00	223.00	−0.49	0.08	672.00	29.46	18.45	26.64	25.45	43.90	54.91	52.08	56.10	47.92	45.09	29.02	14.73	20.98	35.27	64.29	56.25	50.00	35.71	36.16	20.09	26.34	17.41	53.57	43.75	62.50	46.43	23.21	20.54	32.59	23.66	46.88	56.25	55.80	53.13
Ca-CTV	K10	0.22	0.01	0.43	52.10	0.50	0.38	0.44	0.44	217.00	223.00	−0.48	0.08	672.00	28.42	17.86	27.38	26.34	44.20	54.76	53.72	55.80	46.28	45.24	29.91	14.73	20.54	34.82	64.73	55.36	50.45	35.27	35.71	20.09	26.34	17.86	53.57	44.20	62.05	46.43	19.64	18.75	35.27	26.34	45.98	61.61	54.91	54.02
Ca-CTV	K18	0.22	0.02	0.44	54.13	0.50	0.38	0.44	0.44	216.00	223.00	−0.49	0.08	672.00	28.57	18.30	26.93	26.19	44.49	54.76	53.13	55.51	46.88	45.24	29.91	15.18	20.09	34.82	64.73	54.91	50.00	35.27	36.16	20.09	26.34	17.41	53.57	43.75	62.50	46.43	19.64	19.64	34.38	26.34	45.98	60.71	54.02	54.02
Ca-CTV	K20	0.25	0.07	0.47	57.75	0.51	0.38	0.45	0.43	217.00	223.00	−0.49	0.07	672.00	28.87	18.75	26.64	25.74	44.49	54.61	52.38	55.51	47.62	45.39	28.57	15.18	20.09	36.16	64.73	56.25	48.66	35.27	36.16	20.54	25.89	17.41	53.57	43.30	62.05	46.43	21.88	20.54	33.93	23.66	45.54	57.59	55.80	54.46
Ca-CTV	K22	0.25	0.07	0.46	54.78	0.50	0.38	0.44	0.43	217.00	223.00	−0.47	0.08	672.00	29.32	18.75	26.64	25.30	44.05	54.61	51.93	55.95	48.07	45.39	28.57	15.18	20.98	35.27	63.84	56.25	49.55	36.16	36.16	20.54	26.34	16.96	53.13	43.30	62.50	46.88	23.21	20.54	32.59	23.66	46.88	56.25	55.80	53.13
Ca-CTV	K23	0.25	0.07	0.46	55.03	0.50	0.38	0.44	0.43	216.00	223.00	−0.49	0.08	672.00	29.46	18.45	26.64	25.45	43.90	54.91	52.08	56.10	47.92	45.09	29.02	14.73	20.98	35.27	64.29	56.25	50.00	35.71	36.16	20.09	26.34	17.41	53.57	43.75	62.50	46.43	23.21	20.54	32.59	23.66	46.88	56.25	55.80	53.13
Ca-CTV	AR2	0.25	0.07	0.47	58.61	0.51	0.38	0.44	0.42	217.00	223.00	−0.48	0.08	672.00	29.02	18.60	26.79	25.60	44.20	54.61	52.38	55.80	47.62	45.39	28.57	14.73	20.54	36.16	64.73	56.70	49.11	35.27	36.16	20.54	25.89	17.41	53.57	43.30	62.05	46.43	22.32	20.54	33.93	23.21	45.54	57.14	56.25	54.46
Ca-CTV	N1	0.25	0.07	0.47	59.20	0.50	0.38	0.44	0.43	217.00	223.00	−0.45	0.08	672.00	28.87	18.60	26.79	25.74	44.35	54.61	52.53	55.65	47.47	45.39	28.57	14.29	20.98	36.16	64.73	57.14	49.55	35.27	36.16	20.54	25.89	17.41	53.57	43.30	62.05	46.43	21.88	20.98	33.48	23.66	45.54	57.14	55.36	54.46
Ca-CTV	P2	0.26	0.08	0.47	54.12	0.50	0.38	0.44	0.42	216.00	223.00	−0.49	0.08	672.00	29.46	18.30	26.93	25.30	43.60	54.76	52.23	56.40	47.77	45.24	29.02	14.73	20.98	35.27	64.29	56.25	50.00	35.71	36.16	20.09	26.34	17.41	53.57	43.75	62.50	46.43	23.21	20.09	33.48	23.21	46.43	56.70	56.70	53.57
Ca-CTV	P3	0.23	0.02	0.43	51.63	0.50	0.38	0.44	0.44	217.00	223.00	−0.47	0.08	672.00	28.13	18.01	27.38	26.49	44.49	54.61	53.87	55.51	46.13	45.39	29.46	14.73	20.54	35.27	64.73	55.80	50.00	35.27	35.71	20.09	26.34	17.86	53.57	44.20	62.05	46.43	19.20	19.20	35.27	26.34	45.54	61.61	54.46	54.46
Ca-CTV	P4	0.23	0.03	0.44	51.68	0.50	0.38	0.44	0.44	217.00	223.00	−0.48	0.08	672.00	28.27	18.01	27.38	26.34	44.35	54.61	53.72	55.65	46.28	45.39	29.91	14.73	20.54	34.82	64.73	55.36	50.45	35.27	35.71	20.09	26.34	17.86	53.57	44.20	62.05	46.43	19.20	19.20	35.27	26.34	45.54	61.61	54.46	54.46
Ca-CTV	P13	0.23	0.03	0.44	51.68	0.50	0.38	0.44	0.44	217.00	223.00	−0.48	0.08	672.00	28.27	18.01	27.38	26.34	44.35	54.61	53.72	55.65	46.28	45.39	29.91	14.73	20.54	34.82	64.73	55.36	50.45	35.27	35.71	20.09	26.34	17.86	53.57	44.20	62.05	46.43	19.20	19.20	35.27	26.34	45.54	61.61	54.46	54.46
Ca-CTV	TP4	0.22	0.03	0.43	57.22	0.50	0.38	0.44	0.46	217.00	223.00	−0.38	0.08	672.00	28.13	18.75	26.79	26.34	45.09	54.46	53.13	54.91	46.88	45.54	29.46	14.29	20.54	35.71	65.18	56.25	50.00	34.82	34.38	21.43	27.23	16.96	51.34	44.20	61.61	48.66	20.54	20.54	32.59	26.34	46.88	58.93	53.13	53.13
Ca-CTV	TP5	0.22	0.02	0.43	53.11	0.50	0.37	0.43	0.44	216.00	223.00	−0.47	0.08	672.00	28.72	18.15	27.23	25.89	44.05	54.61	53.13	55.95	46.88	45.39	29.91	15.18	20.54	34.38	64.29	54.91	50.45	35.71	36.16	19.64	26.79	17.41	53.57	44.20	62.95	46.43	20.09	19.64	34.38	25.89	45.98	60.27	54.46	54.02
Ca-CTV	TP9	0.22	0.00	0.42	59.06	0.50	0.38	0.44	0.46	217.00	223.00	−0.48	0.08	672.00	28.42	18.60	26.64	26.34	44.94	54.76	52.98	55.06	47.02	45.24	29.91	15.18	20.09	34.82	64.73	54.91	50.00	35.27	35.71	20.09	26.34	17.86	53.57	44.20	62.05	46.43	19.64	20.54	33.48	26.34	45.98	59.82	53.13	54.02
Ca-CTV	CTV-B	0.23	0.03	0.44	53.98	0.50	0.38	0.44	0.45	216.00	223.00	−0.49	0.08	672.00	28.42	18.45	26.79	26.34	44.79	54.76	53.13	55.21	46.88	45.24	29.46	15.18	20.09	35.27	64.73	55.36	49.55	35.27	36.16	20.09	26.34	17.41	53.57	43.75	62.50	46.43	19.64	20.09	33.93	26.34	45.98	60.27	53.57	54.02
Ca-CTV	ML12	0.23	0.07	0.46	51.90	0.49	0.38	0.44	0.48	217.00	223.00	−0.49	0.08	672.00	28.13	18.60	26.64	26.64	45.24	54.76	53.27	54.76	46.73	45.24	29.02	14.29	21.88	34.82	63.84	56.70	50.89	36.16	35.71	19.64	26.34	18.30	54.02	44.64	62.05	45.98	19.64	21.88	31.70	26.79	46.43	58.48	51.34	53.57
Ca-CTV	ML-13	0.25	0.11	0.48	52.69	0.50	0.38	0.44	0.43	217.00	223.00	−0.50	0.08	672.00	29.02	18.30	27.23	25.45	43.75	54.46	52.68	56.25	47.32	45.54	29.46	14.73	20.98	34.82	64.29	55.80	50.45	35.71	36.61	20.09	25.89	17.41	54.02	43.30	62.50	45.98	20.98	20.09	34.82	24.11	45.09	58.93	55.80	54.91
Ca-CTV	HB1	0.24	0.09	0.48	51.94	0.49	0.38	0.44	0.46	217.00	223.00	−0.50	0.08	672.00	28.57	18.60	26.49	26.34	44.94	54.91	52.83	55.06	47.17	45.09	29.46	13.84	21.43	35.27	64.73	56.70	50.89	35.27	35.71	20.09	25.89	18.30	54.02	44.20	61.61	45.98	20.54	21.88	32.14	25.45	45.98	57.59	52.68	54.02
Ca-CTV	NZRB-M17	0.24	0.09	0.48	55.63	0.51	0.38	0.44	0.42	217.00	223.00	−0.47	0.07	672.00	29.17	19.20	26.64	25.00	44.20	54.17	51.64	55.80	48.36	45.83	29.02	15.63	20.09	35.27	64.29	55.36	49.11	35.71	35.71	20.09	26.34	17.86	53.57	44.20	62.05	46.43	22.77	21.88	33.48	21.88	44.64	55.36	56.25	55.36
Cr-CTV	Kpg3	0.25	0.07	0.46	55.11	0.50	0.38	0.44	0.43	216.00	223.00	−0.48	0.08	672.00	29.46	18.60	26.64	25.30	43.90	54.76	51.93	56.10	48.07	45.24	29.02	14.73	20.98	35.27	64.29	56.25	50.00	35.71	36.16	20.54	26.34	16.96	53.13	43.30	62.50	46.88	23.21	20.54	32.59	23.66	46.88	56.25	55.80	53.13

Cr-CTV		0.2	0.0	0.4	53.	0.5	0.3	0.4	0.4	216.	223.	−0.	0.0	672.	28.	18.	26.	26.	44.	54.	53.	55.	46.	45.	29.	15.	20.	35.	64.	55.	49.	35.	36.	20.	26.	17.	53.	43.	62.	46.	19.	20.	33.	26.	45.	60.	53.	54.
	K2	3	4	4	44	0	8	4	5	00	00	49	8	00	27	60	79	34	94	61	13	06	88	39	46	18	09	27	73	36	55	27	16	09	34	41	57	75	50	43	20	54	93	34	54	27	13	46
Cr-CTV		0.2	0.0	0.4	54.	0.5	0.3	0.4	0.4	216.	223.	−0.	0.0	672.	28.	18.	26.	26.	44.	54.	52.	55.	47.	45.	29.	15.	19.	34.	64.	54.	49.	35.	36.	20.	26.	17.	53.	43.	62.	46.	19.	20.	33.	26.	45.	60.	53.	54.
	K8	2	2	4	61	0	8	4	5	00	00	50	7	00	57	60	64	19	79	76	83	21	17	24	91	63	64	82	73	46	55	27	16	09	34	41	57	75	50	43	64	09	93	34	98	27	57	02
Cr-CTV		0.2	0.0	0.4	54.	0.5	0.3	0.4	0.4	216.	223.	−0.	0.0	672.	28.	18.	27.	26.	44.	54.	53.	55.	46.	45.	29.	15.	20.	34.	64.	54.	50.	35.	36.	19.	26.	17.	53.	44.	62.	46.	19.	19.	34.	26.	45.	60.	54.	54.
	K11	2	1	3	57	0	7	4	4	00	00	48	8	00	57	15	08	19	35	76	27	65	73	24	91	18	09	82	73	91	00	27	16	64	79	41	57	20	95	43	64	64	38	34	98	71	02	02
Cr-CTV		0.2	0.0	0.4	57.	0.5	0.3	0.4	0.4	217.	223.	−0.	0.0	672.	29.	18.	26.	25.	44.	54.	52.	55.	47.	45.	29.	14.	20.	35.	64.	56.	49.	35.	36.	20.	25.	17.	53.	43.	62.	46.	22.	20.	33.	23.	45.	57.	56.	54.
	K13	5	7	7	90	0	8	4	2	00	00	49	8	00	17	60	79	45	05	61	23	95	77	39	02	73	54	71	73	25	55	27	16	54	89	41	57	30	05	43	32	54	93	21	54	14	25	46
Cr-CTV		0.2	0.0	0.4	54.	0.5	0.3	0.4	0.4	216.	223.	−0.	0.0	672.	28.	18.	26.	26.	44.	54.	52.	55.	47.	45.	29.	15.	20.	34.	64.	54.	50.	35.	35.	20.	25.	17.	53.	43.	61.	46.	19.	20.	33.	26.	45.	60.	53.	54.
	K16	2	2	4	85	0	8	4	5	00	00	49	8	00	42	60	64	34	94	76	98	06	02	24	91	18	09	82	73	91	00	27	71	54	89	86	57	75	61	43	64	09	93	34	98	27	57	02
Cr-CTV		0.2	0.0	0.4	57.	0.5	0.3	0.4	0.4	217.	223.	−0.	0.0	672.	29.	18.	26.	25.	44.	54.	52.	55.	47.	45.	29.	14.	20.	35.	64.	56.	49.	35.	36.	20.	25.	17.	53.	43.	62.	46.	22.	20.	33.	23.	45.	57.	56.	54.
	K17	5	7	7	90	0	8	4	2	00	00	49	8	00	17	60	79	45	05	61	23	95	77	39	02	73	54	71	73	25	55	27	16	54	89	41	57	30	05	43	32	54	93	21	54	14	25	46
Cr-CTV		0.2	0.0	0.4	54.	0.5	0.3	0.4	0.4	216.	223.	−0.	0.0	672.	29.	18.	26.	25.	44.	54.	51.	55.	48.	45.	28.	15.	20.	35.	63.	56.	49.	36.	36.	20.	25.	16.	53.	42.	62.	46.	23.	20.	32.	24.	47.	56.	55.	52.
	K24	4	6	6	09	0	8	4	3	00	00	52	7	00	32	75	49	45	20	76	93	80	07	24	57	18	98	27	84	25	55	16	16	98	89	96	13	86	05	88	21	09	59	11	32	70	80	68
Cr-CTV		0.2	0.0	0.4	48.	0.5	0.3	0.4	0.4	218.	223.	−0.	0.0	672.	28.	17.	27.	26.	44.	54.	54.	55.	45.	45.	29.	14.	20.	35.	64.	55.	50.	35.	35.	19.	26.	18.	54.	45.	61.	45.	19.	18.	36.	25.	45.	61.	55.	54.
	K27	1	2	3	58	0	8	4	3	00	00	46	7	00	13	71	68	49	20	61	17	80	83	39	46	73	54	27	73	80	00	27	27	64	34	75	02	09	61	98	64	75	16	45	09	61	80	91
Cr-CTV		0.2	0.0	0.4	52.	0.5	0.3	0.4	0.4	217.	223.	−0.	0.0	672.	28.	17.	27.	26.	44.	54.	54.	55.	45.	45.	29.	14.	20.	35.	64.	55.	50.	35.	35.	19.	26.	18.	53.	45.	62.	46.	19.	18.	35.	26.	45.	61.	54.	54.
	K30	2	2	3	10	0	8	4	4	00	00	47	8	00	13	71	53	64	35	76	17	65	83	24	46	73	54	27	73	80	00	27	27	64	79	30	57	09	05	43	64	75	27	34	98	61	91	02
Cr-CTV		0.2	0.0	0.4	57.	0.5	0.3	0.4	0.4	217.	223.	−0.	0.0	672.	29.	18.	26.	25.	44.	54.	52.	55.	47.	45.	29.	14.	20.	35.	64.	56.	49.	35.	36.	20.	25.	17.	53.	43.	62.	46.	22.	20.	33.	23.	45.	57.	56.	54.
	K31	5	7	7	90	0	8	4	2	00	00	49	8	00	17	60	79	45	05	61	23	95	77	39	02	73	54	71	73	25	55	27	16	54	89	41	57	30	05	43	32	54	93	21	54	14	25	46
Cr-CTV		0.2	0.0	0.4	53.	0.5	0.3	0.4	0.4	216.	223.	−0.	0.0	672.	28.	18.	26.	26.	44.	54.	52.	55.	47.	45.	29.	15.	20.	34.	64.	54.	50.	35.	36.	20.	25.	17.	53.	43.	62.	46.	19.	19.	34.	26.	45.	60.	54.	54.
	K33	3	3	4	36	0	8	4	4	00	00	51	7	00	57	45	79	19	64	76	98	36	02	24	91	18	09	82	73	91	00	27	16	54	89	41	57	30	05	43	64	64	38	34	98	71	02	02
Cr-CTV		0.2	0.0	0.4	53.	0.5	0.3	0.4	0.4	216.	223.	−0.	0.0	672.	28.	18.	27.	26.	44.	54.	53.	55.	46.	45.	29.	15.	20.	35.	64.	55.	49.	35.	36.	20.	26.	17.	53.	43.	62.	46.	20.	19.	34.	25.	45.	60.	54.	54.
	K37	3	2	4	36	0	8	4	4	00	00	47	8	00	42	15	23	19	35	61	42	65	58	39	02	18	54	27	29	80	55	71	16	09	34	41	57	75	50	43	09	20	82	89	98	71	91	02
Cr-CTV		0.2	0.0	0.4	53.	0.5	0.3	0.4	0.4	217.	223.	−0.	0.0	672.	28.	18.	27.	26.	44.	54.	53.	55.	46.	45.	29.	15.	20.	35.	64.	55.	49.	35.	36.	20.	26.	17.	53.	43.	62.	46.	19.	19.	34.	26.	45.	61.	54.	54.
	K38	2	1	3	54	0	8	4	4	00	00	48	8	00	42	15	08	34	49	76	42	51	58	24	46	18	09	27	73	36	55	27	16	09	34	41	57	75	50	43	64	20	82	34	98	16	46	02
Cr-CTV		0.2	0.0	0.4	56.	0.5	0.3	0.4	0.4	217.	223.	−0.	0.0	672.	28.	18.	26.	26.	45.	54.	53.	54.	46.	45.	29.	15.	20.	34.	64.	55.	50.	35.	36.	19.	26.	17.	54.	44.	62.	45.	18.	20.	33.	27.	45.	60.	51.	54.
	K39	4	7	7	82	0	8	4	7	00	00	48	8	00	13	60	64	64	24	76	27	76	73	24	46	18	54	82	29	36	00	71	16	64	34	86	02	20	50	98	75	98	04	23	98	27	79	02
Cr-CTV		0.2	0.0	0.4	50.	0.5	0.3	0.4	0.4	217.	223.	−0.	0.0	672.	27.	18.	27.	26.	44.	54.	54.	55.	45.	45.	29.	14.	20.	35.	64.	56.	50.	35.	35.	20.	26.	17.	53.	43.	62.	46.	18.	19.	35.	26.	45.	62.	54.	54.
	K40	3	4	5	11	0	8	4	5	00	00	44	8	00	83	01	53	64	64	46	17	36	83	54	02	29	98	71	73	70	00	27	71	54	34	41	13	75	05	88	75	20	27	79	54	05	02	46
Cr-CTV		0.2	0.0	0.4	53.	0.5	0.3	0.4	0.4	216.	223.	−0.	0.0	672.	29.	18.	26.	25.	43.	54.	52.	56.	47.	45.	29.	14.	21.	34.	63.	56.	50.	36.	36.	20.	25.	17.	53.	43.	62.	46.	23.	19.	33.	24.	47.	57.	56.	52.
	K41	4	5	5	29	0	8	4	2	00	00	52	8	00	46	30	79	45	75	91	23	25	77	09	02	73	43	82	84	25	45	16	16	54	89	41	57	30	05	43	21	64	04	11	32	14	25	68
Cr-CTV		0.2	0.0	0.4	57.	0.5	0.3	0.4	0.4	217.	223.	−0.	0.0	672.	29.	18.	27.	24.	43.	54.	51.	56.	48.	45.	28.	15.	20.	35.	63.	55.	49.	36.	35.	20.	26.	17.	53.	44.	62.	46.	23.	20.	34.	21.	44.	55.	57.	55.
	K42	5	8	7	81	1	8	4	1	00	00	49	7	00	17	90	08	85	75	02	93	25	07	98	57	63	54	27	84	80	11	16	71	09	34	86	57	20	05	43	21	98	38	43	64	80	59	36
Cr-CTV	ARP	0.2	0.0	0.4	52.	0.5	0.3	0.4	0.4	217.	223.	−0.	0.0	672.	28.	17.	27.	26.	44.	54.	54.	55.	45.	45.	29.	14.	20.	35.	64.	55.	50.	35.	35.	19.	26.	18.	53.	45.	62.	46.	19.	18.	35.	26.	45.	61.	54.	54.
	1	2	2	3	10	0	8	4	4	00	00	47	8	00	13	71	53	64	35	76	17	65	83	24	46	73	54	27	73	80	00	27	27	64	79	30	57	09	05	43	64	75	27	34	98	61	91	02
Cr-CTV		0.2	0.0	0.4	57.	0.5	0.3	0.4	0.4	217.	223.	−0.	0.0	672.	29.	18.	26.	25.	43.	54.	52.	56.	47.	45.	28.	14.	21.	35.	63.	56.	50.	36.	36.	20.	26.	17.	53.	43.	62.	46.	23.	20.	33.	23.	46.	56.	56.	53.
	AR13	4	4	4	48	0	8	4	2	00	00	49	8	00	46	30	93	30	60	76	23	40	77	24	57	73	43	27	84	70	00	16	16	09	34	41	57	75	50	43	66	09	04	21	88	25	70	13
Cr-CTV	AG2	0.2	0.0	0.4	56.	0.5	0.3	0.4	0.4	217.	223.	−0.	0.0	672.	29.	18.	26.	24.	43.	54.	51.	56.	48.	45.	28.	15.	20.	35.	63.	55.	49.	36.	36.	20.	26.	17.	53.	43.	62.	46.	25.	19.	33.	21.	46.	55.	58.	53.
	6	7	9	7	43	1	8	4	0	00	00	48	8	00	91	45	93	70	15	61	64	85	36	39	57	63	54	27	84	80	11	16	16	09	34	41	57	75	50	43	00	64	93	43	43	36	93	57
Cr-CTV	AG2	0.2	−0.	0.4	52.	0.5	0.3	0.4	0.4	216.	223.	−0.	0.0	672.	28.	18.	27.	25.	43.	54.	53.	56.	46.	45.	29.	15.	20.	33.	63.	54.	50.	36.	35.	20.	26.	17.	53.	44.	62.	46.	20.	18.	34.	25.	46.	60.	55.	53.
	8	1	02	1	89	0	8	4	3	00	00	48	8	00	87	01	23	89	90	76	13	10	88	24	91	63	54	93	84	46	45	16	71	09	34	86	57	20	05	43	98	30	82	89	88	71	80	13
Cr-CTV		0.2	0.0	0.4	50.	0.5	0.3	0.4	0.4	217.	223.	−0.	0.0	672.	28.	18.	27.	26.	44.	54.	53.	55.	46.	45.	29.	14.	20.	35.	64.	56.	50.	35.	36.	20.	26.	16.	53.	43.	62.	46.	19.	20.	34.	25.	45.	60.	54.	54.
	MB7	3	3	4	30	0	8	4	5	00	00	46	8	00	27	30	23	19	49	46	42	51	58	54	02	29	98	71	73	70	00	27	16	54	34	96	13	30	50	88	64	09	38	89	54	27	02	46
Cr-CTV	Mnp	0.2	0.0	0.4	50.	0.4	0.3	0.4	0.4	217.	223.	−0.	0.0	672.	28.	18.	27.	26.	44.	54.	53.	55.	46.	45.	29.	14.	21.	34.	64.	56.	50.	35.	36.	20.	26.	17.	53.	43.	62.	46.	18.	19.	34.	26.	45.	61.	53.	54.
	1	3	4	5	24	9	8	3	5	00	00	47	8	00	13	01	53	34	35	46	87	65	13	54	46	29	43	82	29	25	89	71	16	09	34	41	57	75	50	43	75	64	82	79	54	61	57	46
Cr-CTV	Mnp	0.2	0.0	0.4	54.	0.5	0.3	0.4	0.4	217.	223.	−0.	0.0	672.	28.	18.	26.	26.	44.	54.	52.	55.	47.	45.	29.	15.	20.	35.	64.	55.	49.	35.	36.	20.	26.	17.	53.	43.	62.	46.	20.	20.	33.	25.	45.	59.	54.	54.
	2	3	2	3	64	0	8	4	5	00	00	48	8	00	57	45	79	19	64	76	98	36	02	24	46	18	09	27	73	36	55	27	16	09	34	41	57	75	50	43	09	09	93	89	98	82	02	02
Cr-CTV	Mnp	0.2	0.0	0.4	54.	0.5	0.3	0.4	0.4	217.	223.	−0.	0.0	672.	28.	18.	26.	26.	44.	54.	53.	55.	46.	45.	29.	15.	20.	35.	64.	55.	49.	35.	35.	20.	26.	17.	53.	44.	61.	46.	19.	19.	34.	26.	45.	60.	54.	54.
	3	2	1	3	06	0	8	4	5	00	00	46	8	00	13	45	93	49	94	61	42	06	58	39	46	18	09	27	73	36	55	27	27	54	34	86	13	20	61	88	64	64	38	34	98	71	02	02
Cr-CTV		0.2	0.0	0.4	53.	0.5	0.3	0.4	0.4	217.	223.	−0.	0.0	672.	29.	19.	26.	25.	44.	54.	51.	55.	48.	45.	29.	14.	20.	35.	64.	56.	50.	35.	36.	20.	26.	17.	53.	43.	62.	46.	21.	22.	32.	22.	44.	55.	54.	55.
	P10	4	9	8	96	0	8	4	4	00	00	49	8	00	17	05	64	15	20	32	79	80	21	68	46	29	98	27	73	25	45	27	16	09	34	41	57	75	50	43	88	77	59	77	64	36	46	36
Cr-CTV	KAT	0.2	0.0	0.4	52.	0.5	0.3	0.4	0.4	217.	223.	−0.	0.0	672.	28.	18.	27.	26.	44.	54.	53.	55.	46.	45.	29.	14.	20.	34.	64.	55.	50.	35.	35.	20.	26.	17.	53.	44.	62.	46.	20.	19.	34.	25.	45.	60.	55.	54.
	5	3	2	3	64	0	8	4	3	00	00	48	8	00	72	01	23	04	05	76	27	95	73	24	91	73	54	82	73	36	45	27	71	09	34	86	57	20	05	43	54	20	82	45	98	27	36	02
Cr-CTV	KAT	0.2	0.0	0.4	51.	0.5	0.3	0.4	0.4	217.	223.	−0.	0.0	672.	28.	17.	27.	26.	44.	54.	53.	55.	46.	45.	29.	14.	20.	34.	64.	55.	50.	35.	35.	20.	26.	17.	53.	44.	62.	46.	19.	18.	35.	26.	45.	61.	54.	54.
	6	3	3	4	71	0	8	4	4	00	00	48	8	00	42	86	38	34	20	76	72	80	28	24	91	73	54	82	73	36	45	27	71	09	34	86	57	20	05	43	64	75	27	34	98	61	91	02
Cr-CTV		0.2	0.0	0.4	50.	0.4	0.3	0.4	0.4	217.	223.	−0.	0.0	672.	28.	18.	26.	26.	44.	54.	53.	55.	46.	45.	29.	14.	21.	34.	64.	56.	50.	35.	36.	19.	26.	17.	54.	44.	62.	45.	19.	20.	33.	26.	45.	59.	52.	54.
	B165	3	6	6	84	9	8	3	7	00	00	48	8	00	27	30	93	49	79	76	42	21	58	24	46	29	43	82	29	25	89	71	16	64	34	86	02	20	50	98	20	98	04	79	98	82	23	02
Cr-CTV	179.0	0.2	0.0	0.4	51.	0.5	0.3	0.4	0.4	217.	223.	−0.	0.0	672.	28.	17.	27.	26.	44.	55.	53.	55.	46.	44.	29.	14.	20.	34.	64.	55.	50.	35.	36.	19.	25.	17.	54.	43.	62.	45.	19.	19.	34.	26.	45.	61.	54.	54.
	0	2	2	3	24	0	8	4	5	00	00	52	8	00	57	86	08	49	35	06	57	65	43	94	91	73	54	82	73	36	45	27	61	64	89	86	46	75	50	54	20	20	82	79	98	61	02	02
Cr-CTV		0.2	0.0	0.4	50.	0.5	0.3	0.4	0.4	217.	223.	−0.	0.0	672.	28.	18.	26.	26.	44.	54.	53.	55.	46.	45.	29.	14.	20.	35.	64.	55.	50.	35.	36.	20.	26.	17.	53.	43.	62.	46.	19.	20.	33.	25.	45.	59.	53.	54.
	A18	5	7	6	61	0	8	4	6	00	00	48	8	00	27	60	93	19	79	46	13	21	88	54	46	73	54	27	73	80	00	27	16	09	34	41	57	75	50	43	20	98	93	89	09	82	13	91
Cr-CTV	FS70	0.2	0.0	0.4	51.	0.4	0.3	0.4	0.4	217.	223.	−0.	0.0	672.	27.	18.	27.	26.	44.	54.	53.	55.	46.	45.	29.	14.	21.	34.	64.	56.	50.	35.	36.	20.	26.	17.	53.	43.	62.	46.	18.	20.	34.	27.	45.	61.	52.	54.
	1-VT	3	2	3	53	9	8	3	6	00	00	50	8	00	98	15	38	49	64	46	87	36	13	54	46	29	43	82	29	25	89	71	16	09	34	41	57	75	50	43	30	09	38	23	54	61	68	46
Cr-CTV	FS70	0.2	0.0	0.4	51.	0.4	0.3	0.4	0.4	217.	223.	−0.	0.0	672.	29.	17.	27.	25.	42.	54.	52.	57.	47.	45.	30.	14.	20.	34.	64.	54.	50.	35.	36.	20.	25.	17.	54.	43.	62.	45.	21.	17.	36.	24.	45.	60.	58.	54.
	1-T30	3	8	7	29	9	8	3	1	00	00	50	8	00	61	56	53	30	86	91	83	14	17	09	36	73	54	38	73	91	89	27	61	09	89	41	02	30	50	98	88	86	16	11	98	27	04	02
Cr-CTV	FS70	0.2	0.0	0.4	54.	0.4	0.3	0.4	0.4	217.	223.	−0.	0.0	672.	29.	18.	27.	24.	43.	54.	51.	56.	48.	45.	30.	14.	20.	33.	64.	54.	51.	35.	36.	20.	26.	17.	53.	43.	62.	46.	21.	21.	34.	22.	44.	57.	55.	55.
	1-T36	4	8	7	55	8	8	3	3	00	00	49	7	00	46	60	23	70	30	17	93	70	07	83	80	29	98	93	73	91	79	27	16	09	34	41	57	75	50	43	43	43	38	77	20	14	80	80

Cr-CTV	FS70	0.2	0.0	0.4	51.	0.4	0.3	0.4	0.4	217.	223.	−0.	0.0	672.	27.	18.	27.	26.	44.	54.	53.	55.	46.	45.	29.	14.	21.	34.	64.	56.	50.	35.	36.	20.	26.	17.	53.	43.	62.	46.	18.	20.	34.	27.	45.	61.	52.	54.
	3-VT	3	2	3	53	9	8	3	6	00	00	50	8	00	98	15	38	49	64	46	87	36	13	54	46	29	43	82	29	25	89	71	16	09	34	41	57	75	50	43	30	09	38	23	54	61	68	46
Cr-CTV	FS70	0.2	0.0	0.4	51.	0.4	0.3	0.4	0.4	217.	223.	−0.	0.0	672.	29.	18.	27.	25.	43.	54.	52.	56.	47.	45.	30.	14.	20.	34.	64.	54.	50.	35.	36.	20.	25.	17.	54.	43.	62.	45.	21.	19.	34.	24.	45.	58.	56.	54.
	3-T30	4	8	7	61	9	8	3	2	00	00	50	8	00	61	01	08	30	30	91	38	70	62	09	36	73	54	38	73	91	89	27	61	09	89	41	02	30	50	98	88	20	82	11	98	93	70	02
Cr-CTV	FS70	0.2	0.0	0.4	54.	0.4	0.3	0.4	0.4	217.	223.	−0.	0.0	672.	29.	18.	27.	24.	43.	54.	52.	56.	47.	45.	30.	14.	20.	34.	64.	55.	51.	35.	36.	20.	26.	17.	53.	43.	62.	46.	21.	21.	34.	22.	44.	57.	55.	55.
	3-T36	3	7	6	21	9	8	3	3	00	00	49	7	00	32	60	23	85	45	17	08	55	92	83	36	29	98	38	73	36	34	27	16	09	34	41	57	75	50	43	43	43	38	77	20	14	80	80
Cs-CTV		0.2	0.1	0.4	57.	0.5	0.3	0.4	0.4	217.	223.	−0.	0.0	672.	29.	19.	26.	24.	44.	54.	51.	55.	48.	45.	29.	15.	20.	34.	64.	54.	49.	35.	36.	20.	26.	17.	53.	43.	62.	46.	22.	21.	33.	22.	44.	55.	55.	55.
	D5	4	0	8	03	0	8	4	3	00	00	49	8	00	32	20	64	85	05	17	49	95	51	83	46	63	09	82	29	91	55	71	16	09	34	41	57	75	50	43	32	88	48	32	64	80	80	36
Cs-CTV		0.2	0.0	0.4	51.	0.5	0.3	0.4	0.4	217.	223.	−0.	0.0	672.	28.	18.	27.	26.	44.	54.	53.	55.	46.	45.	29.	14.	20.	34.	64.	55.	50.	35.	35.	20.	26.	17.	53.	44.	62.	46.	19.	19.	34.	26.	45.	60.	54.	54.
	D6	3	3	4	91	0	8	4	5	00	00	48	8	00	42	15	08	34	49	76	42	51	58	24	91	73	54	82	73	36	45	27	71	09	34	86	57	20	05	43	64	64	38	34	98	71	02	02
Cs-CTV		0.2	0.1	0.4	57.	0.5	0.3	0.4	0.4	217.	223.	−0.	0.0	672.	29.	19.	26.	24.	44.	54.	51.	55.	48.	45.	29.	15.	20.	34.	64.	54.	49.	35.	36.	20.	26.	17.	53.	43.	62.	46.	22.	21.	33.	22.	44.	55.	55.	55.
	D7	4	0	8	03	0	8	4	3	00	00	49	8	00	32	20	64	85	05	17	49	95	51	83	46	63	09	82	29	91	55	71	16	09	34	41	57	75	50	43	32	88	48	32	64	80	80	36
Cs-CTV		0.2	0.0	0.4	51.	0.5	0.3	0.4	0.4	217.	223.	−0.	0.0	672.	28.	18.	27.	26.	44.	54.	53.	55.	46.	45.	29.	14.	20.	34.	64.	55.	50.	35.	35.	20.	26.	17.	53.	44.	62.	46.	19.	19.	34.	26.	45.	61.	54.	54.
	D8	3	2	3	91	0	8	4	5	00	00	48	8	00	27	01	23	49	49	76	72	51	28	24	91	73	54	82	73	36	45	27	71	09	34	86	57	20	05	43	20	20	82	79	98	61	02	02
Cs-CTV		0.2	0.0	0.4	56.	0.5	0.3	0.4	0.4	217.	223.	−0.	0.0	672.	29.	19.	26.	24.	43.	54.	51.	56.	48.	45.	29.	15.	20.	34.	63.	55.	49.	36.	36.	20.	26.	17.	53.	43.	62.	46.	22.	21.	33.	21.	44.	55.	56.	55.
	D10	4	9	8	93	0	8	4	2	00	00	48	8	00	32	20	79	70	90	02	49	10	51	98	02	63	54	82	84	36	55	16	16	09	34	41	57	75	50	43	77	88	48	88	64	36	25	36
Cs-CTV		0.2	0.0	0.4	57.	0.5	0.3	0.4	0.4	217.	223.	−0.	0.0	672.	29.	19.	26.	25.	44.	54.	51.	55.	48.	45.	29.	15.	20.	34.	64.	54.	49.	35.	36.	20.	25.	17.	53.	43.	62.	46.	21.	21.	33.	22.	44.	56.	55.	55.
	D11	4	9	8	90	0	8	4	3	00	00	51	8	00	17	35	49	00	35	17	49	65	51	83	46	63	09	82	29	91	55	71	16	54	89	41	57	30	05	43	88	88	48	77	64	25	36	36
Cs-CTV		0.2	0.0	0.4	57.	0.5	0.3	0.4	0.4	217.	223.	−0.	0.0	672.	29.	19.	26.	24.	44.	54.	51.	55.	48.	45.	29.	15.	20.	34.	64.	54.	49.	35.	36.	20.	26.	17.	53.	43.	62.	46.	22.	22.	33.	21.	44.	54.	55.	55.
	D12	4	9	8	35	0	8	4	3	00	00	49	8	00	46	35	49	70	05	17	19	95	81	83	46	63	09	82	29	91	55	71	16	09	34	41	57	75	50	43	77	32	04	88	64	91	80	36
Cs-CTV		0.2	0.0	0.4	54.	0.5	0.3	0.4	0.4	216.	223.	−0.	0.0	672.	29.	18.	26.	25.	43.	54.	52.	56.	47.	45.	29.	14.	20.	35.	64.	56.	50.	35.	35.	20.	26.	17.	53.	44.	62.	46.	23.	20.	33.	23.	46.	56.	56.	53.
	D13	5	6	6	74	0	8	4	2	00	00	46	8	00	46	30	93	30	60	76	23	40	77	24	02	73	98	27	29	25	00	71	71	09	79	41	13	20	50	88	66	09	04	21	88	25	70	13
Cs-CTV		0.2	0.0	0.4	52.	0.5	0.3	0.4	0.4	217.	223.	−0.	0.0	672.	28.	18.	27.	26.	44.	54.	53.	55.	46.	45.	29.	14.	20.	34.	64.	55.	50.	35.	35.	20.	26.	17.	53.	44.	62.	46.	20.	19.	34.	25.	45.	60.	54.	54.
	D14	3	2	3	33	0	8	4	4	00	00	48	8	00	57	01	23	19	20	76	42	80	58	24	91	73	54	82	73	36	45	27	71	09	34	86	57	20	05	43	09	20	82	89	98	71	91	02
Cs-CTV		0.2	0.0	0.4	54.	0.5	0.4	0.4	0.4	216.	223.	−0.	0.0	672.	27.	18.	26.	26.	45.	54.	53.	54.	46.	45.	29.	14.	20.	34.	64.	55.	50.	35.	34.	20.	25.	19.	53.	45.	60.	46.	19.	20.	33.	26.	45.	60.	53.	54.
	D15	2	2	3	89	0	0	5	5	00	00	48	8	00	98	45	79	79	24	76	57	76	43	24	91	73	54	82	73	36	45	27	38	54	89	20	57	09	27	43	64	09	93	34	98	27	57	02
Cs-CTV		0.2	0.0	0.4	58.	0.5	0.3	0.4	0.4	217.	223.	−0.	0.0	672.	29.	18.	26.	25.	44.	54.	52.	55.	47.	45.	28.	14.	20.	36.	64.	56.	49.	35.	36.	20.	25.	16.	53.	42.	62.	46.	22.	20.	33.	23.	45.	57.	56.	54.
	AR16	5	7	7	14	1	8	4	2	00	00	50	8	00	17	60	79	45	05	61	23	95	77	39	57	73	54	16	73	70	11	27	61	54	89	96	57	86	50	43	32	54	93	21	54	14	25	46
Cs-CTV		0.2	0.0	0.4	50.	0.4	0.3	0.4	0.4	216.	223.	−0.	0.0	672.	28.	18.	27.	25.	44.	54.	53.	55.	46.	45.	29.	14.	21.	34.	64.	56.	50.	35.	36.	20.	25.	16.	53.	42.	62.	46.	20.	19.	34.	25.	45.	60.	54.	54.
	MB3	3	4	4	25	9	8	4	4	00	00	50	8	00	57	30	23	89	20	46	13	80	88	54	46	29	43	82	29	25	89	71	16	98	89	96	13	86	05	88	09	64	38	89	98	27	46	02
Cs-CTV		0.2	0.0	0.4	54.	0.5	0.3	0.4	0.4	216.	223.	−0.	0.0	672.	29.	18.	26.	25.	43.	54.	52.	56.	47.	45.	29.	14.	20.	35.	64.	56.	50.	35.	36.	20.	26.	17.	53.	43.	62.	46.	23.	20.	33.	23.	46.	56.	56.	53.
	P7	6	8	7	12	0	8	4	2	00	00	49	8	00	46	30	93	30	60	76	23	40	77	24	02	73	98	27	29	25	00	71	16	09	34	41	57	75	50	43	21	09	48	21	43	70	70	57
Cs-CTV		0.2	0.0	0.4	53.	0.5	0.3	0.4	0.4	217.	223.	−0.	0.0	672.	29.	19.	26.	25.	44.	54.	51.	55.	48.	45.	29.	14.	20.	35.	64.	56.	50.	35.	36.	20.	26.	17.	53.	43.	62.	46.	21.	22.	32.	22.	44.	55.	54.	55.
	P14	4	9	8	96	0	8	4	4	00	00	49	8	00	17	05	64	15	20	32	79	80	21	68	46	29	98	27	73	25	45	27	16	09	34	41	57	75	50	43	88	77	59	77	64	36	46	36
Cs-CTV	KAT	0.2	0.0	0.4	52.	0.5	0.3	0.4	0.4	217.	223.	−0.	0.0	672.	28.	18.	27.	26.	44.	54.	53.	55.	46.	45.	29.	14.	20.	34.	64.	55.	50.	35.	35.	20.	26.	17.	53.	44.	62.	46.	20.	19.	34.	25.	45.	60.	54.	54.
	1	3	3	4	82	0	8	4	4	00	00	48	8	00	57	01	23	19	20	76	42	80	58	24	91	73	54	82	73	36	45	27	71	09	34	86	57	20	05	43	09	20	82	89	98	71	91	02
Cs-CTV	KAT	0.2	0.0	0.4	52.	0.5	0.3	0.4	0.4	217.	223.	−0.	0.0	672.	28.	18.	27.	26.	44.	54.	53.	55.	46.	45.	29.	14.	20.	34.	64.	55.	50.	35.	35.	20.	26.	17.	53.	44.	62.	46.	20.	19.	34.	25.	45.	60.	55.	54.
	2	3	2	3	64	0	8	4	3	00	00	48	8	00	72	01	23	04	05	76	27	95	73	24	91	73	54	82	73	36	45	27	71	09	34	86	57	20	05	43	54	20	82	45	98	27	36	02
Cs-CTV	KAT	0.2	0.0	0.4	52.	0.4	0.3	0.4	0.4	217.	223.	−0.	0.0	672.	28.	18.	27.	25.	43.	54.	53.	56.	46.	45.	30.	14.	20.	34.	64.	54.	50.	35.	35.	20.	26.	17.	53.	44.	62.	46.	20.	19.	34.	25.	45.	60.	55.	54.
	3	3	2	3	51	9	8	4	3	00	00	48	8	00	87	01	23	89	90	76	13	10	88	24	36	73	54	38	73	91	89	27	71	09	34	86	57	20	05	43	54	20	82	45	98	27	36	02
Cs-CTV	KAT	0.2	0.0	0.4	52.	0.5	0.3	0.4	0.4	217.	223.	−0.	0.0	672.	28.	18.	27.	26.	44.	54.	53.	55.	46.	45.	29.	14.	20.	34.	64.	55.	50.	35.	35.	20.	26.	17.	53.	44.	62.	46.	20.	19.	34.	25.	45.	60.	55.	54.
	4	3	2	3	64	0	8	4	3	00	00	48	8	00	72	01	23	04	05	76	27	95	73	24	91	73	54	82	73	36	45	27	71	09	34	86	57	20	05	43	54	20	82	45	98	27	36	02
Cs-CTV	KAT	0.2	0.1	0.4	57.	0.5	0.3	0.4	0.4	217.	223.	−0.	0.0	672.	29.	19.	26.	24.	44.	54.	51.	55.	48.	45.	29.	16.	19.	34.	64.	54.	49.	35.	36.	20.	26.	17.	53.	43.	62.	46.	22.	21.	33.	22.	44.	55.	55.	55.
	7	4	0	8	05	1	8	4	3	00	00	50	7	00	32	35	49	85	20	17	34	80	66	83	46	07	64	82	29	46	11	71	16	09	34	41	57	75	50	43	32	88	48	32	64	80	80	36
Cs-CTV	CTV-	0.2	0.0	0.4	51.	0.5	0.3	0.4	0.4	217.	223.	−0.	0.0	672.	28.	18.	26.	26.	44.	54.	53.	55.	46.	45.	29.	14.	20.	34.	64.	55.	50.	35.	35.	20.	26.	17.	53.	44.	62.	46.	19.	20.	33.	26.	45.	60.	53.	54.
	N	2	0	2	18	0	8	4	6	00	00	48	8	00	27	30	93	49	79	76	42	21	58	24	91	73	54	82	73	36	45	27	71	09	34	86	57	20	05	43	20	09	93	79	98	71	13	02
Cs-CTV	DKG	0.2	0.0	0.4	56.	0.5	0.4	0.4	0.4	216.	223.	−0.	0.0	672.	28.	18.	26.	26.	45.	54.	53.	54.	46.	45.	29.	14.	20.	34.	64.	55.	50.	35.	33.	20.	25.	19.	53.	45.	59.	46.	20.	20.	33.	25.	45.	58.	54.	54.
	1	1	2	3	24	0	0	5	4	00	00	47	8	00	13	75	79	34	09	46	13	91	88	54	91	73	54	82	73	36	45	27	93	98	89	20	13	09	82	88	54	54	93	00	54	93	46	46
Cs-CTV		0.2	0.0	0.4	54.	0.5	0.3	0.4	0.4	217.	223.	−0.	0.0	672.	29.	18.	27.	25.	43.	54.	52.	56.	47.	45.	29.	13.	21.	35.	64.	57.	50.	35.	35.	20.	26.	17.	53.	44.	62.	46.	22.	21.	33.	22.	45.	56.	55.	54.
	TP1	3	7	6	08	0	8	4	3	00	00	48	8	00	02	45	08	45	90	46	53	10	47	54	02	84	43	71	73	14	45	27	71	09	34	86	57	20	05	43	32	43	48	77	09	25	80	91
Cs-CTV		0.2	0.0	0.4	50.	0.4	0.3	0.4	0.4	216.	223.	−0.	0.0	672.	28.	18.	27.	26.	44.	54.	53.	55.	46.	45.	29.	14.	21.	34.	64.	56.	50.	35.	36.	20.	25.	16.	53.	42.	62.	46.	19.	19.	34.	26.	45.	60.	54.	54.
	TP3	3	3	4	63	9	8	4	4	00	00	50	8	00	42	30	23	04	35	46	27	65	73	54	46	29	43	82	29	25	89	71	16	98	89	96	13	86	05	88	64	64	38	34	98	71	02	02
Cs-CTV		0.2	0.0	0.4	55.	0.5	0.3	0.4	0.4	217.	223.	−0.	0.0	672.	28.	19.	26.	26.	45.	54.	52.	54.	47.	45.	29.	14.	20.	34.	64.	55.	50.	35.	35.	20.	26.	18.	53.	44.	61.	46.	20.	22.	31.	25.	46.	57.	51.	53.
	TP6	3	3	4	60	0	8	4	7	00	00	47	7	00	42	05	19	34	39	76	53	61	47	24	46	73	98	82	29	80	45	71	27	09	34	30	57	64	61	43	54	32	25	89	43	14	79	57
Cs-CTV		0.2	0.0	0.4	55.	0.5	0.3	0.4	0.4	216.	223.	−0.	0.0	672.	28.	18.	26.	26.	45.	54.	52.	54.	47.	45.	29.	16.	19.	34.	64.	54.	49.	35.	36.	20.	26.	17.	53.	43.	62.	46.	19.	20.	33.	26.	46.	60.	53.	53.
	TP7	2	2	3	85	1	8	4	5	00	00	48	7	00	42	75	49	34	09	76	83	91	17	24	46	07	64	82	29	46	11	71	16	09	34	41	57	75	50	43	64	09	48	79	43	27	13	57
Cs-CTV		0.2	0.0	0.4	53.	0.4	0.3	0.4	0.4	217.	223.	−0.	0.0	672.	28.	18.	27.	26.	44.	54.	53.	55.	46.	45.	30.	14.	20.	34.	64.	54.	50.	35.	35.	20.	26.	17.	53.	44.	62.	46.	19.	19.	35.	25.	45.	61.	54.	54.
	TP10	2	2	3	82	9	8	4	4	00	00	49	8	00	57	01	38	04	05	61	42	95	58	39	36	73	54	38	73	91	89	27	71	09	34	86	57	20	05	43	64	20	27	89	54	16	91	46
Cs-CTV		0.2	0.0	0.4	53.	0.5	0.3	0.4	0.4	217.	223.	−0.	0.0	672.	29.	18.	26.	25.	43.	54.	52.	56.	47.	45.	29.	13.	21.	35.	64.	57.	50.	35.	36.	20.	25.	17.	54.	43.	62.	45.	21.	21.	33.	23.	45.	56.	55.	54.
	TP11	4	8	7	65	0	8	4	3	00	00	51	7	00	17	45	93	45	90	61	38	10	62	39	02	84	43	71	73	14	45	27	61	09	89	41	02	30	50	98	88	43	48	21	09	70	36	91
Cs-CTV		0.2	0.0	0.4	56.	0.5	0.3	0.4	0.4	217.	223.	−0.	0.0	672.	28.	18.	26.	26.	44.	54.	53.	55.	46.	45.	29.	15.	20.	34.	64.	54.	50.	35.	35.	20.	26.	17.	53.	44.	62.	46.	20.	19.	34.	25.	45.	60.	54.	54.
	TP12	3	2	3	58	0	8	4	4	00	00	48	8	00	57	30	93	19	49	76	13	51	88	24	91	18	09	82	73	91	00	27	71	09	34	86	57	20	05	43	09	64	38	89	98	27	46	02
Cs-CTV		0.2	0.0	0.4	52.	0.5	0.3	0.4	0.4	216.	223.	−0.	0.0	672.	28.	18.	26.	26.	44.	54.	53.	55.	46.	45.	30.	14.	20.	34.	65.	54.	50.	34.	35.	20.	26.	17.	53.	44.	62.	46.	19.	20.	33.	26.	45.	60.	53.	54.
	F5	3	3	4	87	0	8	4	5	00	00	49	8	00	57	30	79	34	64	91	13	36	88	09	36	73	09	82	18	91	45	82	71	09	34	86	57	20	05	43	64	09	93	34	98	27	57	02
Cs-CTV		0.2	0.1	0.4	55.	0.5	0.3	0.4	0.4	217.	223.	−0.	0.0	672.	29.	19.	26.	24.	43.	54.	51.	56.	48.	45.	29.	15.	20.	34.	63.	55.	49.	36.	35.	20.	26.	17.	53.	44.	62.	46.	24.	21.	33.	20.	45.	54.	57.	54.
	O2	4	0	8	35	0	8	4	1	00	00	50	7	00	61	05	79	55	60	17	34	40	66	83	02	63	54	82	84	36	55	16	71	09	34	86	57	20	05	43	11	43	48	98	09	46	59	91
Cs-CTV		0.2	0.0	0.4	52.	0.5	0.3	0.4	0.4	216.	223.	−0.	0.0	672.	28.	18.	26.	26.	44.	54.	52.	55.	47.	45.	29.	15.	20.	34.	64.	54.	50.	35.	36.	20.	26.	17.	53.	43.	62.	46.	19.	20.	33.	26.	45.	60.	53.	54.
	O6	3	4	4	82	0	8	4	5	00	00	49	8	00	57	45	79	19	64	76	98	36	02	24	91	18	09	82	73	91	00	27	16	09	34	41	57	75	50	43	64	09	93	34	98	27	57	02

Cs-CTV		0.2	0.0	0.4	56.	0.4	0.3	0.4	0.4	217.	223.	−0.	0.0	672.	29.	18.	26.	25.	43.	54.	51.	56.	48.	45.	29.	14.	21.	34.	63.	56.	50.	36.	36.	20.	26.	16.	53.	43.	62.	46.	23.	20.	32.	23.	46.	55.	55.	53.
	O7	4	5	5	92	9	7	3	3	00	00	50	8	00	76	45	79	00	45	76	79	55	21	24	02	29	88	82	84	70	89	16	61	09	34	96	57	30	95	43	66	98	14	21	88	36	80	13
Cs-CTV		0.2	0.0	0.4	55.	0.5	0.3	0.4	0.4	215.	223.	−0.	0.0	672.	28.	18.	27.	26.	44.	54.	53.	55.	46.	45.	29.	15.	20.	34.	64.	54.	50.	35.	35.	20.	26.	17.	53.	44.	62.	46.	20.	19.	34.	25.	45.	60.	54.	54.
	P2	2	3	4	64	0	8	4	4	00	00	47	8	00	57	30	08	04	35	61	13	65	88	39	91	18	09	82	73	91	00	27	71	09	79	41	13	20	50	88	09	64	38	89	98	27	46	02
Cs-CTV		0.2	0.0	0.4	56.	0.5	0.3	0.4	0.4	216.	223.	−0.	0.0	672.	29.	18.	26.	25.	43.	54.	52.	56.	47.	45.	29.	14.	20.	35.	64.	56.	50.	35.	35.	19.	26.	18.	54.	44.	62.	45.	24.	20.	33.	21.	45.	55.	57.	54.
	P9	6	8	7	36	0	8	4	1	00	00	48	7	00	61	30	93	15	45	76	08	55	92	24	02	73	98	27	29	25	00	71	71	64	34	30	02	64	05	98	11	54	48	88	98	36	59	02
Cs-CTV		0.2	0.0	0.4	54.	0.5	0.3	0.4	0.4	217.	223.	−0.	0.0	672.	29.	18.	26.	25.	43.	54.	52.	56.	47.	45.	28.	14.	21.	35.	63.	56.	50.	36.	36.	20.	26.	17.	53.	43.	62.	46.	23.	20.	32.	23.	46.	56.	55.	53.
	Q4	5	6	6	86	0	8	4	3	00	00	49	8	00	32	45	79	45	90	76	23	10	77	24	57	73	43	27	84	70	00	16	16	09	34	41	57	75	50	43	21	54	59	66	88	25	80	13
Cs-CTV		0.2	0.0	0.4	53.	0.5	0.3	0.4	0.4	218.	223.	−0.	0.0	672.	28.	18.	26.	26.	44.	54.	53.	55.	46.	45.	29.	14.	20.	35.	64.	55.	50.	35.	35.	20.	25.	17.	53.	43.	61.	46.	19.	20.	33.	26.	45.	60.	53.	54.
	Q8	3	2	4	26	0	8	4	5	00	00	49	8	00	27	45	79	49	94	76	27	06	73	24	46	73	54	27	73	80	00	27	71	54	89	86	57	75	61	43	64	09	93	34	98	27	57	02
Cs-CTV		0.2	0.0	0.4	52.	0.5	0.3	0.4	0.4	217.	223.	−0.	0.0	672.	28.	18.	26.	26.	44.	54.	53.	55.	46.	45.	29.	14.	20.	34.	64.	55.	50.	35.	35.	20.	26.	17.	53.	44.	62.	46.	19.	20.	33.	26.	45.	59.	53.	54.
	Q14	3	3	4	82	0	8	4	6	00	00	48	8	00	42	45	79	34	79	76	13	21	88	24	91	73	54	82	73	36	45	27	71	09	34	86	57	20	05	43	64	54	48	34	98	82	13	02
Cs-CTV		0.2	0.0	0.4	50.	0.4	0.3	0.4	0.4	217.	223.	−0.	0.0	672.	28.	18.	27.	25.	43.	54.	53.	56.	46.	45.	29.	14.	21.	34.	64.	55.	51.	35.	36.	19.	26.	16.	53.	43.	63.	46.	19.	20.	34.	26.	45.	60.	53.	54.
	S4	3	2	3	97	9	7	3	5	00	00	48	8	00	57	01	53	89	90	46	42	10	58	54	91	29	43	38	29	80	34	71	61	64	79	96	57	75	39	43	20	09	38	34	54	71	57	46
Cs-CTV	CT14	0.2	0.0	0.4	51.	0.4	0.3	0.4	0.4	217.	223.	−0.	0.0	672.	28.	18.	26.	26.	44.	54.	52.	55.	47.	45.	29.	14.	22.	34.	63.	56.	51.	36.	36.	19.	26.	17.	54.	44.	62.	45.	19.	22.	31.	25.	45.	57.	51.	54.
	A	3	6	6	47	9	8	3	8	00	00	50	9	00	27	90	79	04	94	32	83	06	17	68	02	29	32	38	39	70	34	61	16	64	34	86	02	20	50	98	64	77	70	89	54	59	34	46
Cs-CTV	CT11	0.2	0.0	0.4	57.	0.4	0.3	0.4	0.4	217.	223.	−0.	0.0	672.	28.	18.	26.	26.	44.	54.	52.	55.	47.	45.	29.	14.	21.	34.	64.	56.	50.	35.	36.	19.	26.	17.	54.	44.	62.	45.	20.	21.	32.	25.	45.	58.	53.	54.
	A	3	4	5	19	9	8	3	6	00	00	48	8	00	72	45	79	04	49	76	83	51	17	24	46	29	43	82	29	25	89	71	16	64	34	86	02	20	50	98	54	43	59	45	98	04	13	02
Cs-CTV		0.2	0.0	0.4	52.	0.4	0.3	0.4	0.4	217.	223.	−0.	0.0	672.	28.	19.	26.	25.	44.	54.	52.	55.	47.	45.	29.	14.	21.	34.	63.	56.	50.	36.	35.	19.	26.	17.	53.	44.	62.	46.	20.	23.	31.	24.	45.	55.	52.	54.
	AT-1	3	8	7	63	9	8	3	7	00	00	45	8	00	57	05	64	74	79	32	38	21	62	68	02	29	88	82	84	70	89	16	71	64	79	86	57	64	50	43	98	21	25	55	54	80	23	46
Cs-CTV		0.2	0.0	0.4	53.	0.5	0.3	0.4	0.4	217.	223.	−0.	0.0	672.	27.	18.	27.	26.	44.	54.	53.	55.	46.	45.	29.	14.	20.	35.	64.	56.	50.	35.	36.	20.	26.	16.	53.	43.	62.	46.	18.	20.	33.	27.	45.	61.	52.	54.
	VT	3	2	3	92	0	8	4	6	00	00	49	8	00	98	45	08	49	94	46	57	06	43	54	02	73	98	27	29	25	00	71	16	54	34	96	13	30	50	88	75	09	93	23	98	16	68	02
Cs-CTV	NZ-	0.2	0.0	0.4	51.	0.4	0.3	0.4	0.4	217.	223.	−0.	0.0	672.	28.	18.	26.	26.	44.	54.	53.	55.	46.	45.	29.	14.	21.	34.	64.	56.	50.	35.	36.	19.	26.	17.	54.	44.	62.	45.	19.	21.	32.	26.	45.	59.	51.	54.
	B18	3	6	6	50	9	8	3	7	00	00	48	8	00	27	45	79	49	94	76	27	06	73	24	46	29	43	82	29	25	89	71	16	64	34	86	02	20	50	98	20	43	59	79	98	38	79	02
Cs-CTV	108.0	0.2	0.0	0.4	51.	0.5	0.3	0.4	0.4	217.	223.	−0.	0.0	672.	28.	18.	27.	26.	44.	54.	53.	55.	46.	45.	29.	14.	20.	34.	64.	55.	50.	35.	35.	20.	26.	17.	53.	44.	62.	46.	19.	19.	34.	26.	45.	61.	53.	54.
	0	3	3	4	06	0	8	4	5	00	00	48	8	00	27	15	08	49	64	76	57	36	43	24	91	73	54	82	73	36	45	27	71	09	34	86	57	20	05	43	20	64	38	79	98	16	57	02
Cs-CTV	109.0	0.2	0.0	0.4	51.	0.5	0.3	0.4	0.4	217.	223.	−0.	0.0	672.	28.	18.	26.	26.	44.	54.	53.	55.	46.	45.	29.	14.	20.	34.	64.	55.	50.	35.	35.	20.	26.	17.	53.	44.	62.	46.	19.	20.	33.	26.	45.	60.	53.	54.
	0	3	3	4	59	0	8	4	5	00	00	48	8	00	42	30	93	34	64	76	27	36	73	24	91	73	54	82	73	36	45	27	71	09	34	86	57	20	05	43	64	09	93	34	98	27	57	02
Cs-CTV	135.0	0.2	0.0	0.4	51.	0.5	0.3	0.4	0.4	217.	223.	−0.	0.0	672.	28.	18.	27.	26.	44.	54.	53.	55.	46.	45.	29.	14.	20.	34.	64.	55.	50.	35.	35.	20.	26.	17.	53.	44.	62.	46.	19.	19.	34.	26.	45.	61.	54.	54.
	0	2	2	3	77	0	8	4	4	00	00	48	8	00	42	01	23	34	35	76	57	65	43	24	91	73	54	82	73	36	45	27	71	09	34	86	57	20	05	43	64	20	82	34	98	16	46	02
Cs-CTV	142.0	0.2	−0.	0.4	51.	0.4	0.3	0.4	0.4	216.	223.	−0.	0.1	672.	27.	16.	28.	26.	43.	54.	55.	56.	44.	45.	28.	13.	22.	35.	63.	57.	50.	36.	35.	18.	28.	18.	53.	46.	63.	46.	19.	17.	36.	26.	45.	62.	55.	54.
	0	1	04	0	59	9	7	3	3	00	00	41	0	00	83	67	87	64	30	46	51	70	49	54	57	84	32	27	84	59	89	16	27	30	13	30	57	43	39	43	64	86	16	34	98	50	80	02
Cs-CTV	143.0	0.2	0.0	0.4	51.	0.5	0.3	0.4	0.4	217.	223.	−0.	0.0	672.	28.	18.	27.	26.	44.	54.	53.	55.	46.	45.	29.	14.	20.	34.	64.	55.	50.	35.	35.	20.	26.	17.	53.	44.	62.	46.	19.	19.	34.	26.	45.	61.	54.	54.
	0	2	2	3	77	0	8	4	4	00	00	48	8	00	42	01	23	34	35	76	57	65	43	24	91	73	54	82	73	36	45	27	71	09	34	86	57	20	05	43	64	20	82	34	98	16	46	02
Cs-CTV	146.0	0.2	0.0	0.4	54.	0.5	0.3	0.4	0.4	217.	223.	−0.	0.0	672.	28.	18.	26.	26.	44.	54.	53.	55.	46.	45.	29.	15.	20.	35.	64.	55.	49.	35.	35.	20.	26.	17.	53.	44.	62.	46.	19.	20.	33.	26.	45.	60.	53.	54.
	0	2	3	4	63	0	8	4	5	00	00	47	8	00	27	45	79	49	94	76	27	06	73	24	46	18	09	27	73	36	55	27	71	09	34	86	57	20	05	43	64	09	93	34	98	27	57	02
Cs-CTV	159.0	0.2	0.0	0.4	51.	0.5	0.3	0.4	0.4	217.	223.	−0.	0.0	672.	28.	17.	27.	26.	44.	54.	53.	55.	46.	45.	29.	14.	20.	34.	64.	55.	50.	35.	36.	20.	25.	17.	54.	43.	62.	45.	19.	18.	35.	26.	45.	62.	54.	54.
	0	3	2	4	37	0	8	4	4	00	00	51	8	00	42	86	23	49	35	91	72	65	28	09	91	73	54	82	73	36	45	27	16	09	89	86	02	75	05	98	20	75	27	79	98	05	46	02
Cs-CTV	160.0	0.2	0.0	0.4	52.	0.5	0.3	0.4	0.4	217.	223.	−0.	0.0	672.	28.	18.	27.	26.	44.	54.	53.	55.	46.	45.	29.	14.	20.	34.	64.	55.	50.	35.	35.	20.	26.	17.	53.	44.	62.	46.	20.	19.	34.	25.	45.	60.	54.	54.
	0	2	2	3	08	0	8	4	4	00	00	48	8	00	57	01	23	19	20	76	42	80	58	24	91	73	54	82	73	36	45	27	71	09	34	86	57	20	05	43	09	20	82	89	98	71	91	02
Cs-CTV	SYR-	0.2	0.0	0.4	51.	0.4	0.3	0.4	0.4	217.	223.	−0.	0.0	672.	28.	18.	27.	26.	44.	54.	53.	55.	46.	45.	29.	14.	21.	34.	64.	55.	51.	35.	36.	20.	26.	17.	53.	43.	62.	46.	18.	20.	33.	26.	45.	60.	52.	54.
	A2	3	4	5	58	9	8	3	6	00	00	50	8	00	27	30	23	19	49	46	42	51	58	54	91	29	43	38	29	80	34	71	16	09	34	41	57	75	50	43	75	54	93	79	54	71	68	46
Cs-CTV		0.2	0.0	0.4	51.	0.5	0.3	0.4	0.4	217.	223.	−0.	0.0	672.	28.	18.	27.	26.	44.	54.	53.	55.	46.	45.	29.	14.	20.	35.	64.	55.	50.	35.	36.	20.	26.	17.	53.	43.	62.	46.	20.	19.	34.	26.	46.	60.	54.	53.
	T3	3	3	4	63	0	8	4	4	00	00	48	8	00	57	01	08	34	35	91	42	65	58	09	46	73	54	27	73	80	00	27	16	09	34	41	57	75	50	43	09	20	38	34	43	71	46	57
Cs-CTV	FL20	0.2	0.0	0.4	50.	0.4	0.3	0.4	0.4	217.	223.	−0.	0.0	672.	28.	17.	27.	26.	43.	54.	53.	56.	46.	45.	29.	14.	21.	34.	64.	56.	50.	35.	36.	20.	26.	17.	53.	43.	62.	46.	19.	19.	35.	25.	45.	61.	54.	54.
	2-VT	3	4	5	41	9	8	3	4	00	00	48	8	00	42	86	68	04	90	46	72	10	28	54	46	29	43	82	29	25	89	71	16	09	34	41	57	75	50	43	64	20	27	89	54	16	91	46
Cs-CTV	FS67	0.2	0.0	0.4	53.	0.4	0.3	0.4	0.4	217.	223.	−0.	0.0	672.	29.	18.	27.	24.	43.	54.	51.	56.	48.	45.	30.	14.	20.	34.	64.	55.	51.	35.	36.	20.	26.	17.	53.	43.	62.	46.	21.	21.	33.	22.	44.	56.	55.	55.
	4-T36	4	8	7	84	9	8	3	3	00	00	49	7	00	32	75	08	85	60	17	93	40	07	83	36	29	98	38	73	36	34	27	16	09	34	41	57	75	50	43	43	88	93	77	20	70	36	80
	**Aver**	0.2	0.0	0.4	53.	0.5	0.3	0.4	0.4	216.	223.	−0.	0.0	672.	28.	18.	26.	25.	44.	54.	52.	55.	47.	45.	29.	14.	20.	35.	64.	55.	50.	35.	35.	20.	26.	17.	53.	43.	62.	46.	20.	20.	33.	25.	45.	59.	54.	54.
	**age**	3	5	5	88	0	8	4	4	75	00	48	8	00	73	38	99	90	28	63	89	72	11	37	48	79	69	04	51	73	17	49	94	13	30	63	57	93	24	43	77	22	98	04	81	01	75	19
		0.0	0.0	0.0	2.4	0.0	0.0	0.0	0.0			0.0	0.0		0.5	0.4	0.3	0.5	0.5	0.2	0.7	0.5	0.7	0.2	0.5	0.4	0.5	0.4	0.3	0.6	0.6	0.3	0.4	0.3	0.2	0.4	0.3	0.5	0.4	0.3	1.6	1.0	0.9	1.7	0.6	2.2	1.4	0.6
	**SD**	1	3	2	0	1	0	0	2	0.48	0.00	2	0	0.00	1	1	4	9	1	2	7	1	7	2	1	5	2	5	3	7	0	3	1	5	9	0	0	1	3	0	0	5	6	1	3	2	8	3
		0.0	0.0	0.0	0.2	0.0	0.0	0.0	0.0			0.0	0.0		0.0	0.0	0.0	0.0	0.0	0.0	0.0	0.0	0.0	0.0	0.0	0.0	0.0	0.0	0.0	0.0	0.0	0.0	0.0	0.0	0.0	0.0	0.0	0.0	0.0	0.0	0.1	0.0	0.0	0.1	0.0	0.2	0.1	0.0
	**SE**	0	0	0	2	0	0	0	0	0.04	0.00	0	0	0.00	5	4	3	5	5	2	7	5	7	2	5	4	5	4	3	6	5	3	4	3	3	4	3	5	4	3	4	9	9	5	6	0	3	6

Note: Ca-CTV, Cr-CTV, and Cs-CTV denote the CTV isolates originated from *C. aurantifolia*, *C. reticulata*, and *C. sinensis*, respectively. Values within parentheses indicate ± standard errors. A%, U%, C%, and G% represent the overall frequencies of adenine (A), cytosine (C), uracil (U), and guanine (G) nucleotides at the coding sequence of CP gene; A3%, U3%, C3%, and G3% represent the nucleotide frequency at the third codon position; GC and AU denote the percentage of G+C and A+U; GC1, GC2, and GC3 denote the G+C at the first, second, and third synonymous codon positions; GA3, GU3, AU3, and CU3 represent the G+A, G+U, A+U, and C+U percentages at the third codon position; CAI, codon adaptation index, measures the relative addictiveness of the codon usage of a gene toward the codon usage of highly expressed genes; CBI, codon bias index, measures the extent to which a gene uses a subset of optimal codons; Fop, frequency of optimum codons, is the ratio of optimal codons to synonymous codons; ENc, effective number of codons; L_sym, number of synonymous codons; L_aa, number of translatable codons; Gravy, general average hydropathicity; Aromo, frequency of aromatic amino acids in hypothetically translated gene product.

Table S3. Contribution of host-preferred high-frequency codons, virus-preferred high-frequency codons, and other synonymous codons in coat protein of CTV isolates.

AA^{ni}	Ca-CTV							Cr-CTV							Cs-CTV						
	AA%	RSCU$_H$	RSCU$_V$	CHFC$_H$ (per AA) %	CHFC$_V$ (per AA) %	CHFC%	COSC%	AA%	RSCU$_H$	RSCU$_V$	CHFC$_H$ (per AA) %	CHFC$_V$ (per AA) %	CHFC%	COSC%	AA%	RSCU$_H$	RSCU$_V$	CHFC$_H$ (per AA) %	CHFC$_V$ (per AA) %	CHFC%	COSC%
**Leu^{6}**	11.78	3.67	1.54	7.21 (61.17)	3.02 (25.67)	10.23	1.55	11.78	3.82	1.40	7.50 (63.67)	2.75 (23.33)	10.25	1.53	11.81	3.75	1.47	7.38 (62.50)	2.89 (24.50)	10.27	1.53
**Ser^{6}**	4.86	2.27	1.45	1.84 (37.83)	1.17 (24.17)	3.01	1.85	4.83	2.42	1.57	1.95 (40.33)	1.26 (26.17)	3.21	1.62	4.94	2.42	1.53	1.99 (40.33)	1.26 (25.50)	3.25	1.69
**Arg^{6}**	5.75	1.18	2.18	1.13 (19.67)	2.09 (36.33)	3.22	2.53	5.89	1.09	2.21	1.07 (18.17)	2.17 (36.83)	3.24	2.65	5.88	1.12	2.21	1.10 (18.67)	2.17 (36.83)	3.26	2.62
**Ala^{4}**	7.70	1.90	0.00	3.66 (47.50)	0.00 (0.00)	3.66	4.04	7.67	1.86	0.00	3.57 (46.50)	0.00 (0.00)	3.57	4.10	7.49	1.85	0.00	3.47 (46.25)	0.00 (0.00)	3.47	4.03
**Gly^{4}**	7.70	1.62	0.00	3.12 (40.50)	0.00 (0.00)	3.12	4.58	7.56	1.54	0.00	2.91 (38.50)	0.00 (0.00)	2.91	4.65	7.57	1.48	0.00	2.80 (37.00)	0.00 (0.00)	2.80	4.77
**Val^{4}**	6.60	1.34	1.15	2.21 (33.50)	1.90 (28.75)	4.11	2.49	6.53	2.50	0.00	4.08 (62.50)	0.00 (0.00)	4.08	2.45	6.59	2.56	1.07	4.22 (64.00)	1.76 (26.75)	5.98	0.61
**Pro^{4}**	2.20	1.49	2.11	0.82 (37.25)	1.16 (52.75)	1.98	0.22	2.14	1.44	2.30	0.77 (36.00)	1.23 (57.50)	2.00	0.14	2.10	1.38	2.28	0.73 (34.50)	1.20 (57.00)	1.92	0.18
**Thr^{4}**	7.05	1.72	0.00	3.03 (43.00)	0.00 (0.00)	3.03	4.02	7.10	1.86	0.00	3.30 (46.50)	0.00 (0.00)	3.30	3.80	7.25	1.84	0.00	3.33 (46.00)	0.00 (0.00)	3.33	3.91
**Ile^{3}**	2.95	0.00	1.59	0.00 (0.00)	1.57 (53.00)	1.57	1.39	3.07	0.00	1.60	0.00 (0.00)	0.00 (0.00)	0.00	3.07	3.02	0.00	1.65	0.00 (0.00)	1.66 (55.00)	1.66	1.36
**Asp^{2}**	9.37	1.16	0.00	5.43 (58.00)	0.00 (0.00)	5.43	3.94	9.36	1.17	0.00	5.47 (58.50)	0.00 (0.00)	5.47	3.88	9.35	1.16	0.00	5.42 (58.00)	0.00 (0.00)	5.42	3.93
**Lys^{2}**	7.58	0.00	0.00	0.00 (0.00)	1.16 (15.31)	1.16	6.42	7.59	1.16	0.00	4.40 (58.00)	0.00 (0.00)	4.40	3.19	7.59	0.00	1.21	0.00 (0.00)	4.59 (60.50)	4.59	3.00
**Asn^{2}**	6.60	1.48	0.00	4.89 (74.00)	0.00 (0.00)	4.89	1.72	6.66	0.00	1.41	0.00 (0.00)	4.69 (70.50)	4.69	1.96	6.56	0.00	1.41	0.00 (0.00)	4.62 (70.50)	4.62	1.93
**Glu^{2}**	4.07	0.00	1.32	0.00 (0.00)	2.68 (66.00)	2.68	1.38	4.07	0.00	1.26	0.00 (0.00)	2.56 (63.00)	2.56	1.51	4.07	0.00	1.35	0.00 (0.00)	2.75 (67.50)	2.75	1.32
**Tyr^{2}**	3.54	0.00	1.29	0.00 (0.00)	2.28 (64.50)	2.28	1.26	3.56	0.00	1.28	0.00 (0.00)	2.28 (64.00)	2.28	1.28	3.55	1.29	0.00	2.29 (64.50)	0.00 (0.00)	2.29	1.26
**Phe^{2}**	3.14	1.11	0.00	1.74 (55.50)	0.00 (0.00)	1.74	1.40	3.12	1.14	0.00	1.78 (57.00)	0.00 (0.00)	1.78	1.34	3.19	0.00	0.00	0.00 (0.00)	0.00 (0.00)	0.00	3.19
**Gln^{2}**	3.57	0.00	0.00	0.00 (0.00)	0.00 (0.00)	0.00	3.57	3.58	0.00	0.00	0.00 (0.00)	0.00 (0.00)	0.00	3.58	3.57	0.00	0.00	0.00 (0.00)	0.00 (0.00)	0.00	3.57
**His^{2}**	1.36	0.00	1.41	0.00 (0.00)	0.96 (70.50)	0.96	0.40	1.38	0.00	1.38	0.00 (0.00)	0.95 (69.00)	0.95	0.43	1.35	0.00	1.39	0.00 (0.00)	0.94 (69.50)	0.94	0.41
**Cys^{2}**	1.35	0.00	2.00	0.00 (0.00)	1.35(100.00)	1.35	0.00	1.33	0.00	1.95	0.00 (0.00)	1.30 (97.50)	1.30	0.03	1.34	1.95	0.00	1.30 (97.50)	0.00 (0.00)	1.30	0.03
**Trp^{1}**	0.90	0.00	0.00	0.00 (0.00)	0.00 (0.00)	0.00	0.90	0.91	0.00	0.00	0.00 (0.00)	0.00 (0.00)	0.00	0.91	0.94	0.00	0.00	0.00 (0.00)	0.00 (0.00)	0.00	0.94
**Met^{1}**	1.93	0.00	0.00	0.00 (0.00)	0.00 (0.00)	0.00	1.93	1.89	0.00	0.00	0.00 (0.00)	0.00 (0.00)	0.00	1.89	1.85	0.00	0.00	0.00 (0.00)	0.00 (0.00)	0.00	1.85
**Total**	**100**	**18.94**	**16.04**	**35.08 (35.08)**	**19.34 (19.34)**	**54.42**	**45.58**	**100**	**20.00**	**16.36**	**36.80 (36.80)**	**19.20 (19.20)**	**55.99**	**44.01**	**100**	**20.80**	**15.57**	**34.03 (34.03)**	**23.84 (23.84)**	**57.87**	42.13

AA^{ni} represents the three-letter abbreviation code of 20 amino acids with ni kind of synonymous codons; AA% represents the percentage of amino acids in coat protein of CTV. Ca-CTV, Cr-CTV, and Cs-CTV denote the CTV isolates originated from *C. aurantifolia*, *C. reticulata*, and *C. sinensis*, respectively. RSCU$_H$ and RSCU$_V$ denote the total relative synonymous codon usage values of host-preferred high-frequency codons and virus-preferred high-frequency codons, respectively. CHFC$_H$ represents the contribution of host-preferred high-frequency codons, and CHFC$_V$ represents the contribution of virus-preferred high-frequency codons. CHFC denotes total contribution of host-preferred and virus-preferred high-frequency codons (CHFC$_H$+ CHFC$_V$). COSC represents the contribution of other synonymous codons; values within parentheses indicate high-frequency codons per amino acid (AA) contribution percentage.
